# The Arabidopsis NPF7.2 mediates coumarin uptake for root iron acquisition

**DOI:** 10.1111/nph.70993

**Published:** 2026-03-02

**Authors:** Shunsuke Watanabe, Meijie Li, Alice Rossille, Chérhazad Boustani, Kevin Robe, Yuri Kanno, Mitsunori Seo, Christian Dubos

**Affiliations:** ^1^ IPSiM, Univ. Montpellier, CNRS, INRAE, Institut Agro Montpellier France; ^2^ Department of Biological Science, Faculty of Science and Engineering Yasuda Women’s University Hiroshima 731‐0153 Japan; ^3^ RIKEN Center for Sustainable Resource Science Yokohama 230‐0045 Japan; ^4^ Tropical Biosphere Research Center, University of the Ryukyus Okinawa 903‐0213 Japan

**Keywords:** coumarin trafficking, Fe‐mobilizing coumarins, iron acquisition, NPF transporter, root secretion

## Abstract

- Iron (Fe) is a transition metal necessary for achieving essential physiological processes throughout the plant lifecycle. In *Arabidopsis thaliana*, secreting Fe‐mobilizing coumarins (FMC) is a key mechanism enabling roots to acquire nonbioavailable Fe present in soils.
- Here, we unveil the pivotal role played by NRT1/PTR FAMILY 7.2 (NPF7.2) in coumarin secretion by conducting phenotypic analyses of NPF7.2 loss‐of‐function mutants, NPF7.2 expression–localization analyses and direct transport assay of NPF7.2 protein with coumarin compounds using yeast cells.
- NPF7.2 loss‐of‐function impairs coumarin secretion and Fe acquisition in Arabidopsis seedlings, and the corresponding mutants are hypersensitive to Fe deficiency. NPF7.2 protein colocalizes with the main coumarin exporter, PDR9 (PLEIOTROPIC DRUG RESISTANCE PROTEIN 9), in both cortex and epidermis cell layers in root areas where coumarin secretion occurs. A site‐specific accumulation of fraxin, a storage form of fraxetin, in root peripheral tissues is disturbed in the mutants under Fe deficiency. When expressed in yeast cells, NPF7.2 protein has an uptake activity for fraxetin and scopoletin, a major FMC and its precursor, respectively. However, fraxin and scopolin, their storage form, are not transported.
- We propose that NPF7.2 facilitates PDR9‐mediated FMC secretion from the epidermis into the rhizosphere by the cellular loading of coumarins from apoplastic spaces, leading to optimal Fe acquisition.

## Introduction

Iron (Fe), a major micronutrient for most living organisms, plays essential roles in various biological processes (e.g. respiration and photosynthesis) by serving as a cofactor for several metalloproteins (Przybyla‐Toscano *et al*., 2021). Any disturbance in its homeostasis results in cellular defects and thus inhibition of growth. In humans, Fe deficiency is estimated to affect as many as 2 billion people world‐wide (Camaschella, 2015), notably in population in which plant‐based diets predominate. Combating Fe‐deficiency anaemia requires improving Fe content in diets without increasing meat consumption. Biofortification of plants by improving their capacity to absorb and store Fe is one possibility to provide adequate dietary Fe to the growing global population, in a sustainable manner (Ofori *et al*., 2022). Nonetheless, such a strategy greatly benefits from the acquired knowledge on the molecular mechanisms that control Fe homeostasis in plants.

Although Fe is one of the most abundant elements found in soil, it is generally poorly available to plants since it is mainly present in the form of insoluble Fe(III) oxides/hydroxides, in particular at alkaline pH (Colombo *et al*., 2018). To cope with this poor bioavailability, dicot and nongraminaceous monocot plants have evolved an elaborate mechanism to take up, via their root system, Fe from soils (Li *et al*., 2023). It consists of the secretion of protons into the rhizosphere to reduce the pH and increase the solubility of Fe that is then reduced into Fe^2+^, by FERRIC REDUCTION OXIDASES, and taken up inside the root cells by transporters of the ZIP (ZRT/IRT‐LIKE PROTEIN) family. In *Arabidopsis thaliana*, these three steps rely on the activity of AHA2 (proton‐ATPase 2), FERRIC REDUCTION OXIDASE 2 and IRON‐REGULATED TRANSPORTER 1 (IRT1), respectively.

It has emerged that Fe uptake is also promoted by the synthesis and secretion into the rhizosphere of Fe‐mobilizing coumarins (FMCs; Robe *et al*., 2021a). Coumarins are plant specialized metabolites derived from the phenylpropanoid pathway whose biosynthesis relies, at least in part, on FERULOYL‐CoA 6′‐HYDROXYLASE 1 (F6′H1) activity (Kai *et al*., 2008). Coumarins are glycosylated before their storage into the vacuoles and are secreted as aglycones following their deglycosylation by specific β‐GLUCOSIDASES, such as BGLU42 (Zamioudis *et al*., 2014; Wu *et al*., 2022). The two main FMCs are fraxetin and sideretin whose biosynthesis and secretion are induced in response to Fe deficiency and relies on the activity of SCOPOLETIN 8‐HYDROXYLASE and CYP82C4 activity, respectively (Rajniak *et al*., 2018; Siwinska *et al*., 2018; Tsai *et al*., 2018). Sideretin is the main FMC secreted at acidic pH, allowing the reduction of Fe^3+^ into Fe^2+^ before its subsequent uptake by IRT1 (Rajniak *et al*., 2018; Paffrath *et al*., 2023). At alkaline pH, this is fraxetin that is mostly secreted. The way by which fraxetin participates in Fe nutrition at this pH has been a matter of debate for several years (Robe *et al*., 2021b; Li *et al*., 2023). Recently, it was shown that fraxetin form stable complexes with Fe(III) that are directly translocated into the roots via a yet unknown mechanism (Robe *et al*., 2025). Importantly, in addition to promoting plant Fe uptake, coumarin secretion plays also important roles in shaping plant root microbiome and therefore in modulating plant growth and health (Stassen *et al*., 2021).

Coumarin trafficking is a complex and dynamic process where the PLEIOTROPIC DRUG RESISTANCE 9/ARABIDOPSIS THALIANA ATP‐BINDING CASSETTE G37 (PDR9/ABCG37) transporters play a preponderant role (Fourcroy *et al*., 2014). For instance, PDR9 is involved in the root transport of FMCs from the cortex to the epidermal cells and their subsequent secretion into the surrounding media (Robe *et al*., 2021a). Noteworthy, these cell types were recently found to take up scopoletin and fraxetin even when coumarin secretion is strongly stimulated by Fe deficiency (Robe *et al*., 2021a). These findings indicate that cellular coumarin uptake might play an important role in root Fe acquisition. It also supports that FMCs transport also involves additional as yet unidentified transporters (Robe *et al*., 2021a,b).

NITRATE TRANSPORTER1/PEPTIDE TRANSPORTER FAMILY (NPF) proteins form a large family of proton‐dependent bidirectional transporters for nitrate and di‐/tri‐peptides in various plant species (Léran *et al*., 2014). Among the 53 Arabidopsis NPF proteins, several have been shown to play essential roles in the movements of small phytochemicals, such as glucosinolates and plant hormones, indicating that their biochemical and physiological functions are diverse. In addition, some NPFs share substrates with ABC transporters. For instance, the plant hormone abscisic acid is the common substrate of both NPF4.6 and ABCG25 (Kuromori *et al*., 2018). Interestingly, PDR9 is also known to display an indole‐3‐butyric acid transport activity, such as NPF7.3 (Ruzicka *et al*., 2010; Watanabe *et al*., 2020). These observations suggest that NPF proteins could display coumarin transport activities and therefore participate to plant Fe nutrition. Interestingly, among the NPF family members, NRT1/PTR FAMILY 7.2 (*NPF7.2*)*/NRT1.8* has its expression induced in roots in response to Fe deficiency (Colangelo & Guerinot, 2004) and tightly correlated with that of *BGLU42* (Zamioudis *et al*., 2014). These observations indicating that NPF7.2 could be involved in the transport of FMCs in Arabidopsis roots.

In this study, we aimed to determine whether NPF7.2 mediates the transport of coumarins in Arabidopsis roots to improve plant Fe nutrition. We found that NPF7.2 functions as a coumarin import transporter, whose expression is induced in response to Fe deficiency, that is localized at the root plasma membrane of peripheral tissues composed of cortical and epidermal cells. We also found that *npf7.2* loss‐of‐function mutants that are hypersensitive to Fe deficiency can be rescued by exogenous application of fraxetin, a major FMC. Furthermore, the Fe‐deficiency‐dependent secretion of coumarins is inhibited in *npf7.2* mutants when compared to the wild‐type (WT) whereas the endogenous levels of fraxin, a storage form of fraxetin, within the root is increased. Nevertheless, the specific accumulation of fraxin in cortex and epidermis within the zone of the root where coumarin secretion begins is significantly reduced, which may disrupt an optimal fraxin distribution. Last, we found that NPF7.2 protein shows an uptake activity for fraxetin and scopoletin, a precursor of fraxetin. Together, these results suggest that NPF7.2 potentiates the intensive coumarin secretion from epidermal cells towards the rhizosphere via PDR9 by facilitating the cellular loading of coumarins from apoplastic spaces, which leads to the enhancement of plant Fe acquisition.

## Materials and Methods

### Plant materials and growth conditions

Arabidopsis (*Arabidopsis thaliana* (L.) Heynh.) accession Columbia‐0 (Col‐0) was used as the WT for all experiments in this work. Seeds of the *npf7.2* mutants were obtained from the Arabidopsis Biological Resource Center (Ohio State University, Columbus, OH, USA) as follows: *npf7.2‐1* (SALK_024892), *npf7.2‐2* (SAIL_389_D03) and *npf7.2‐3* (GK‐756D01). The other mutants used in this study are *f6′h1‐1* (Kai *et al*., 2008) and *pdr9‐2* (Fourcroy *et al*., 2014). The homozygous *npf7.2* mutants were isolated by PCR genotyping using appropriate primer pairs (Supporting Information Table S1), which were designed by the T‐DNA Primer Design Tool (signal.salk.edu/tdnaprimers.2.html). After the sterilization of seed surfaces with 70% (v/v) ethanol containing 5% (v/v) NaClO and then 70% ethanol (v/v), seeds were sown on ½‐strength Murashige & Skoog medium (½MS) containing 1% (w/v) sucrose and 1.2% (w/v) agar. MES or MOPS (3‐(N‐morpholino)propanesulfonic acid) were added to the media and pH was adjusted with 1 M KOH to 5.7 for acidic media or to 7.0 for alkaline media, respectively. For seedling growth analysis in poorly Fe available conditions, 100 μM FeCl_3_ was added to the media instead of 50 μM Fe(III)‐EDTA. After 3 d stratification at 4°C in the dark, the plates were vertically placed in growth chambers at 22°C under white light with a 16‐h photoperiod (120 μmol photons·m^−2^·s^−1^). For induction of coumarin accumulation and secretion, 7‐d‐old seedlings grown on ½MS containing sufficient Fe‐ethylenediaminetetraacetic acid (EDTA) were transferred to poorly Fe available media containing FeCl_3_ as a sole Fe nutrition at pH 5.7 and 7.0 and then treated for 3 d in the same long‐day conditions as described above, unless otherwise stated. For fraxetin uptake test, 10‐d‐old seedlings grown on ½MS containing sufficient Fe‐EDTA were transferred to Fe‐deficiency media containing 20 μM fraxetin at pH 7.0 and then treated for 1 d in the same long‐day conditions.

### Vector construction and generation of transgenic plants

The primer and synthetic oligonucleotide sequences used for vector constructions in this work are listed in Table S1. All binary vectors generated in this study were validated by sequencing and introduced into *Agrobacterium tumefaciens* (GV3101 strain) by heat shock before transformation of Arabidopsis plants by the floral dip method (Clough & Bent, 1998).

For generating CRISPR/Cas9‐based *NPF7.2* deletion mutants (*npf7.2‐4* and *npf7.2‐5*), guide RNA (gRNA) was designed by Crispr‐P v.2.0 (http://cbi.hzau.edu.cn/CRISPR2/) (Lei *et al*., 2014). The target‐binding gRNA was prepared by annealing two complementary oligos pairs NPF7.2‐gRNA1‐st and NPF7.2‐gRNA1‐cp, NPF7.2‐gRNA2‐st and NPF7.2‐gRNA2‐cp and NPF7.2‐gRNA3‐st and NPF7.2‐gRNA3‐cp. Each fragment was ligated into MultiSite Gateway vectors containing AtU6gRNA, pMR203 (L1‐L4), pMR204 (R4‐R3) and pMR205 (L3‐L2) that were linearized by BbsI restriction enzyme digestion. All gRNAs were assembled by LR reaction in the modified pKAMA‐ITACHI Red (pKIR1) containing attR1‐ccdB‐CmR‐attR2 Gateway cassette (pKIR1‐Gateway). To generate pKIR1‐Gateway, the Gateway cassette was amplified from pDe‐Cas9 by PCR using primers ccdB‐F1 and ccdB‐R1, followed by nested PCR using primers ccdB‐F2 and ccdB‐R2. The final fragment was introduced between SalI and PmeI cloning sites in the CRISPR/Cas9 pKIR1 vector (Addgene plasmid #86409; Tsutsui & Higashiyama, 2017) before WT plants transformation. RED FLUORESCENT PROTEIN (RFP) fluorescence in seeds was used to select primary transformants. Deletion mutants were selected by PCR genotyping using primers NPF7.2g‐F4 and NPF7.2g‐R3 and the obtained DNA fragments were sequenced for sequence confirmation.

For complementation test of *npf7.2‐3*, the 1.6‐kb promoter and the 2.0‐kb whole genomic region of *NPF7.2* (*ProNPF7.2:gNPF7.2*) were amplified by PCR with primers (pE4)‐proNPF7.2‐F and gNPF7.2‐(pE4)‐R. The amplicon was cloned by Gibson assembly reaction into the linear pENTR4 amplified by inverse PCR using primers pE4‐InvF1 and pE4‐InvR1 (proNPF7.2:gNPF7.2‐pENTR4). To insert the sequence of omega translational enhancer (Ω) between the *NPF7.2* promoter and genomic regions, the Ω fragment that was produced by annealing two oligos (proNPF7.2)‐Ω‐(gNPF7.2)‐st and (proNPF7.2)‐Ω‐(gNPF7.2)‐cp was inserted by Gibson assembly into the proNPF7.2:gNPF7.2‐pENTR4 vector linearized by inverse PCR using primers proNPF7.2‐gNPF7.2‐pE4‐InvF and proNPF7.2‐gNPF7.2‐pE4‐InvR (proNPF7.2Ω:gNPF7.2‐pENTR4). The sequence of *ProNPF7.2Ω:gNPF7.2* in the pENTR4 vector was then transferred into pFAST‐R07 (Shimada *et al*., 2010) by LR reaction and used to transform *npf7.2‐3* plants.

For analysing tissue‐specific transcription using reporter genes, the 1.6 kb of *NPF7.2* promoter sequence was amplified by PCR using primers (pE4)‐proNPF7.2‐F and proNPF7.2‐(pE4)‐R and then cloned by Gibson assembly into pENTR4 that was linearized by digesting with NcoI and XhoI. The promoter sequence was transferred into pFAST‐G04 by LR reaction before WT plants transformation.

For direct transport assay of NPF7.2 protein in yeast cells, full‐length cNPF7.2 without the stop codon was amplified from cDNA prepared from Arabidopsis roots by PCR using primers (pE4)‐cNPF7.2‐F2 and cNPF7.2‐(pE4)‐R2. Then, the PCR fragment was cloned into the linear pENTR4, that was linearized by PCR using primers pE4‐InvF1 and pE4‐InvR2, by Gibson assembly (cNPF7.2∆STOP‐pENTR4) and transferred into pB7FWG2 (Karimi *et al*., 2002) to generate a fusion with enhanced *GREEN FLUORESCENT PROTEIN* (GFP) (cNPF7.2‐GFP‐pB7FWG2). The cNPF7.2‐GFP fusion sequence was amplified by PCR using (pE4)‐cNPF7.2‐F1 and GFP‐(pE4)‐R and subcloned into pENTR4 by Gibson assembly reaction into the pENTR4 linearized by inverse PCR using primers pE4‐InvF1 and pE4‐InvR1 (cNPF7.2‐GFP‐pENTR4). To generate the expression vectors, *cNPF7.2* and *cNPF7.2‐GFP* were transferred from cNPF7.2‐pENTR4 and cNPF7.2‐GFP‐pENTR4 into pAG426GAL‐ccdB yeast expression by LR reaction (vector Addgene plasmid #14155; Alberti *et al*., 2007), respectively. The resulting vectors were used for yeast transformation as described below.

### Direct transport assay

In this study, *Saccharomyces cerevisiae* BY4741 strain (*MATa his3Δ1 leu2Δ0 met15Δ0 ura3Δ0*) was used for direct transport assay. Yeast transformation and direct transport assay were performed according to the protocol previously reported with some modifications (Watanabe *et al*., 2022). In brief, yeast cells transformed with the expression vectors were cultivated in synthetic defined (SD) media containing 2% (w/v) glucose and lacking uracil (Ura) at 30°C for 24 h, followed by induction of protein expression for a further 24 h in SD media containing 2% (w/v) galactose and 1% (w/v) raffinose and lacking Ura. After the induction, cells were collected by centrifuging at 5000 g for 5 min and then washed with 20 mM potassium phosphate buffer (KPB) at pH 5.5 three times. The cells were resuspended in the KPB pH 5.5 or pH 7.0 at OD600 = 20. The final suspension was incubated at 25°C for 5 min, followed by the preparation of 500 μl aliquots of the suspension. Transport assays were started by adding 500 μl of a coumarin solution at the indicated final concentrations. Co‐incubation was arrested by centrifugation at 4°C at 20 000 ***g*** for 1 min after 60 min of incubation unless otherwise stated. Following three times washes of cells with KPB pH 7.0, the pellets were incubated in 80% (v/v) methanol at 80°C for 15 min to extract coumarins incorporated in cells. The extraction was clarified by centrifugation at 4°C at 20 000 ***g*** for 5 min. After the evaporation of the supernatants, the precipitations were resuspended in 10% (v/v) methanol solution containing 0.1% formic acid followed by the quantification of coumarins by high‐performance liquid chromatography (HPLC) using the conditions described below.

### Total RNA extraction and quantitative RT‐PCR

Total RNA was prepared from root tissues of 14‐d‐old Arabidopsis seedlings using NucleoSpin RNA Kit (Macherey‐Nagel, Düren, Germany). cDNA was synthesized from 0.5 μg of total RNA by reverse transcription using RevertAid RT Reverse Transcription Kit (Thermo Scientific, Waltham, MA, USA) and was used for quantitative reverse transcription polymerase chain reaction (qRT‐PCR). mRNA levels were quantified using LightCycler® 480 System (Roche). All PCR amplifications contained 1× SYBR Green Master Mix (Takara, Shiga, Japan), 0.2 μM forward and reverse primers, and cDNA in a total volume of 20 μl. *PROTEIN PHOSPHATASE2A SUBUNIT A3* was used as a reference gene (Czechowski *et al*., 2005). The sequence of primers used for qRT‐PCR are listed Table S1.

### Analysis of microarray data

RAW data set of microarray experiments from Dinneny *et al*., (2008) using Arabidopsis roots exposed to Fe deficiency was retrieved from the Gene Expression Omnibus collection (GSE10576; Dinneny *et al*., 2008). Expression levels were normalized according to Watanabe *et al*. (2018) using the R statistical software v.4.2. 1 (R Core Team) and RStudio v.2023.06.2 + 561 (RStudio Team). Transporter genes that are classified into ‘Transporter activity’ (707 genes) in TAIR database (https://www.arabidopsis.org/) were isolated from the gene list. Differentially expressed transporter genes in response to Fe deficiency were identified by one‐way ANOVA with false discovery rate controlled at 5%. Hierarchical clustering analysis with Euclidean distance and complete linkage, and heat map visualization were performed using the pheatmap package in the R software. Gene Ontology (GO) analysis was performed based on ‘Molecular Function’ category in GO annotation on the Gene Ontology Consortium website (http://www.geneontology.org). The enriched GO terms were summarized and visualized using the Revigo software (Supek *et al.*, 2011).

### Measurements of coumarin levels

Root tissues excised from Arabidopsis seedlings were homogenized in 80% (v/v) methanol using TissueLyser II (Qiagen) with metal beads to extract metabolites, followed by overnight incubation at 4°C in the dark. After the filtration of the extraction, the resulting filtrates were vacuum dried and resuspended in 10% (v/v) methanol containing 0.1% (v/v) formic acid, and then subjected to HPLC analysis. Quantification of coumarin compounds (i.e. esculin, esculetin, scopolin, scopoletin, fraxin and fraxetin) was performed using an Agilent LC1220 Infinity II HPLC system according to the protocol previously reported in Robe *et al*. (2021a,b).

### Chlorophylls content analysis

Chlorophylls from four 1‐wk‐old seedlings, or 25 mg of aerial tissues (fresh weight, FW) from 2‐wk‐old seedlings, were extracted in 1 ml 80% acetone in the dark under agitation. The absorbance (*A*) at 663.2 and 646.8 nm was then measured. Total chlorophylls content was assessed using the following equations: Chl*a* + Chl*b* = 7.15*A_663.2_ + 18.71*A_646.8_ and expressed as mg per plant or mg g^−1^ FW.

### Iron content analysis

Fe was extracted from 10 to 30 mg of 2‐wk‐old seedlings (dry weight) by homogenizing in 750 μl of nitric acid (65% (v/v)) and 250 μl of hydrogen peroxide (30% (v/v)). After incubation at room temperature for degassing, the samples were mineralized overnight (*c*. 16 h) on a heat block at 85°C. Samples were then diluted with 4 ml of ultrapure water. Fe content in the samples was quantified by inductively coupled plasma‐optical emission spectrometry (Agilent Technologies, Santa Clara, CA, USA).

### Histochemical GUS analysis

*β‐GLUCURONIDASE* (GUS) staining was carried out according to Watanabe *et al*. (2020). Stained samples were mounted in chloral hydrate after stopping the reaction by 1 h of sequential incubation with ethanol series (50, 70, 95 and 100% (v/v)) and then were observed using a BX61 microscope (Olympus, Tokyo, Japan).

### Confocal microscopy

Fluorescent images were obtained using a confocal laser scanning microscope SP8 (Leica, Solms, Germany). GFP and mCherry were excited at 488 and 561 nm, and the signals were detected between 500 and 550 nm and 610 and 660 nm, respectively. For propidium iodide (Pi) staining, roots were detached from whole seedlings and then stained in 30 μM PI solution. Fluorescence of PI was excited using a 488‐nm laser for excitation, and emission signals were detected between 600 and 650 nm.

Images of cellular coumarins were acquired using an LSM 880 multi‐photon microscope (Zeiss, Oberkochen, Germany) equipped with a Chameleon Ultra II laser (Coherent, Santa Clara, CA, USA). The conditions for image acquisition and spectral deconvolution were based on the previous report (Robe *et al*., 2021a, 2023).

### Statistical analysis

Significant differences between two groups were detected by Student's *t*‐test. Significant differences among three or more groups were detected by ANOVA with *post hoc* Tukey's test. All statistical values were calculated using the statistical software Prism 8 (GraphPad Software Inc.) or RStudio v.4.2.1 (RStudio Team).

## Results

### NPF7.2 loss‐of‐function impairs plant iron nutrition

The Fe‐deficiency response is regulated at the transcriptional level by a complex regulatory network (Gao *et al*., 2020, 2024; Gao & Dubos, 2021; Li *et al*., 2023). In order to identify potential FMC transporters, transcriptomes of roots subjected to Fe deficiency were analysed (Dinneny *et al*., 2008), focusing on genes encoding proteins classified into ‘Transporter activity’ in TAIR. Six hundred eighty‐eight candidates were identified of which 52 and 52 were upregulated and downregulated in response to Fe deficiency, respectively (Fig. S1a,b; Table S2). GO analysis highlighted that these transporters belonged to different families, including NPF (Fig. S1c).

**Fig. 1:**
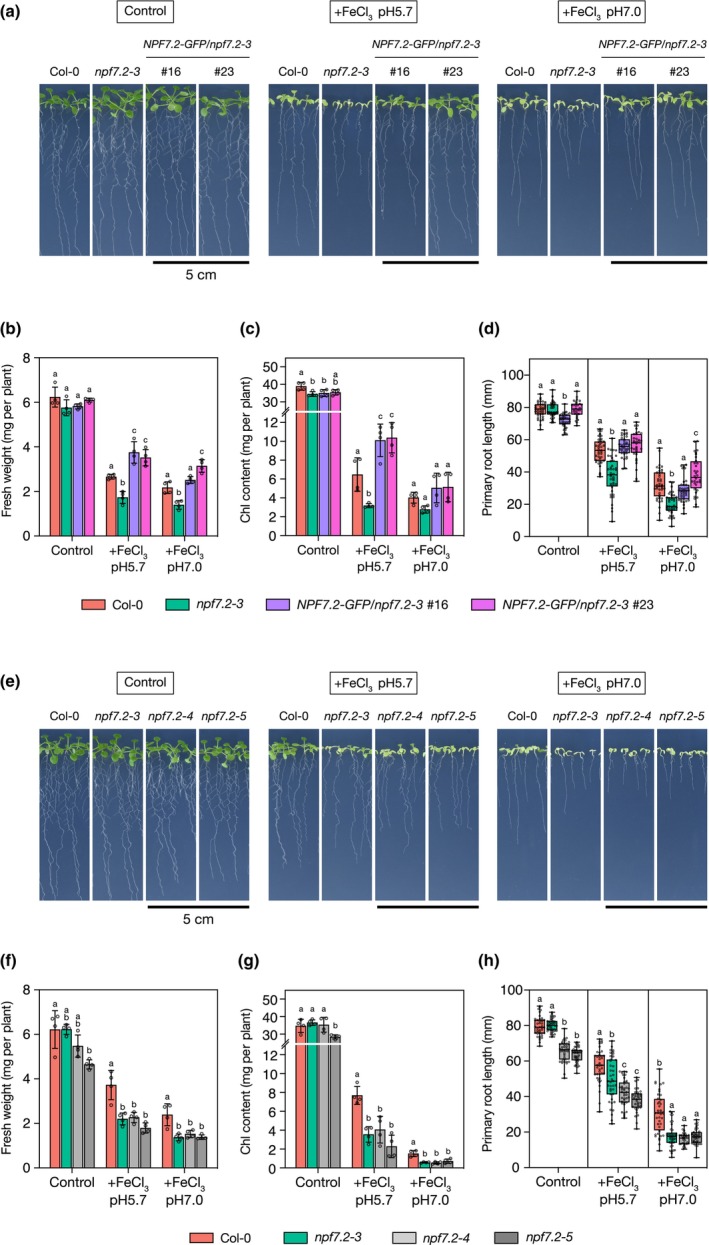
Phenotypic characterization of *npf7.2* loss‐of‐function mutants. (a) Phenotypes of the Arabidopsis wild‐type (Columbia‐0 (Col‐0)), *npf7.2‐3* mutant, and two complemented *npf7.2‐3* mutant lines expressing the *ProNPF7.2:gNPF7.2‐GFP* transgene (*NPF7.2‐GFP/npf7.2‐3*) grown for 1 wk on iron (Fe)‐sufficient (50 μM Fe‐EDTA) conditions or in presence of poorly available Fe (100 μM FeCl_3_). Bars, 5 cm. (b–d) Fresh weight (FW) (*n* = 4) (b), Chl content (*n* = 4) (c) and primary root length (*n* = 32–41) (d) of seedlings described in (a), respectively. (e) Phenotypes of the Col‐0, *npf7.2‐3*, and two deletion mutants of NPF7.2, *npf7.2‐4* and *npf7.2‐5*, grown as in (a). Bars, 5 cm. (f–h) FW (*n* = 4) (f), Chl content (*n* = 4) (g) and primary root length (*n* = 36–42) (h) of seedlings described in (e), respectively. (b–d, f–h) Means within each condition with the same letter are not significantly different according to one‐way ANOVA followed by *post hoc* Tukey test, *P* < 0.05. (b, c, f, g) Error bars show ± SD. Dots represent individual measurements. (d, h) Horizontal bars represent the median, boxes represent the middle 50% of the distribution, whiskers represent the entire spread of the data, and dots represent individual measurements. *NPF7.2*, NRT1/PTR FAMILY 7.2; NPF, NITRATE TRANSPORTER1/PEPTIDE TRANSPORTER FAMILY; EDTA, ethylenediaminetetraacetic acid; *GFP*, *GREEN FLUORESCENT PROTEIN*.

Among the potential candidates, we focused on *NPF7.2* because its expression was associated with MYB72, a transcription factor known to regulate coumarin secretion (Zamioudis *et al*., 2014). Assuming that NPF7.2 plays an important role in coumarin secretion‐based solubilization of rhizospheric Fe, disruption of NPF7.2‐mediated coumarin transport was expected to negatively impact the Fe acquisition activity of Arabidopsis roots. To assess this possibility, we analysed the Fe‐deficiency response of *npf7.2* loss‐of‐function mutants grown in presence of poorly available Fe (i.e. FeCl_3_), whose bioavailability to plants increases upon FMC secretion into the rhizosphere. Three T‐DNA insertion lines (i.e. *npf7.2‐1*, *npf7.2‐2* and *npf7.2‐3*; Fig. S2a–c) together with two CRISPR‐Cas9 lines were analysed (i.e. *npf7.2‐4* and *npf7.2‐5*; Fig. S3). When compared to the WT, all lines exhibited a hypersensitive response to Fe deficiency at both acidic and alkaline pH, including a decreased FW, Chl content and primary root growth similar to that of the *f6′h1‐1* and *pdr9‐2* mutants (Figs 1, S2d–g). Consistent with the growth inhibition, Fe contents were significantly reduced in *npf7*.2 mutants compared with the WT under Fe deficiency (Fig. S4). We then generated *npf7.2‐3* lines expressing the *ProNPF7.2:gNPF7.2‐GFP* transgene. We observed that the growth defects observed in *npf7.2‐3* mutant were rescued, indicating that the *ProNPF7.2:gNPF7.2‐GFP* transgene was functional and sufficient to complement the *npf7.2‐3* mutation (Figs 1a–d, S5).

### npf7.2 Fe‐deficiency hypersensitivity is rescued by exogenous application of fraxetin

**Fig. 2:**
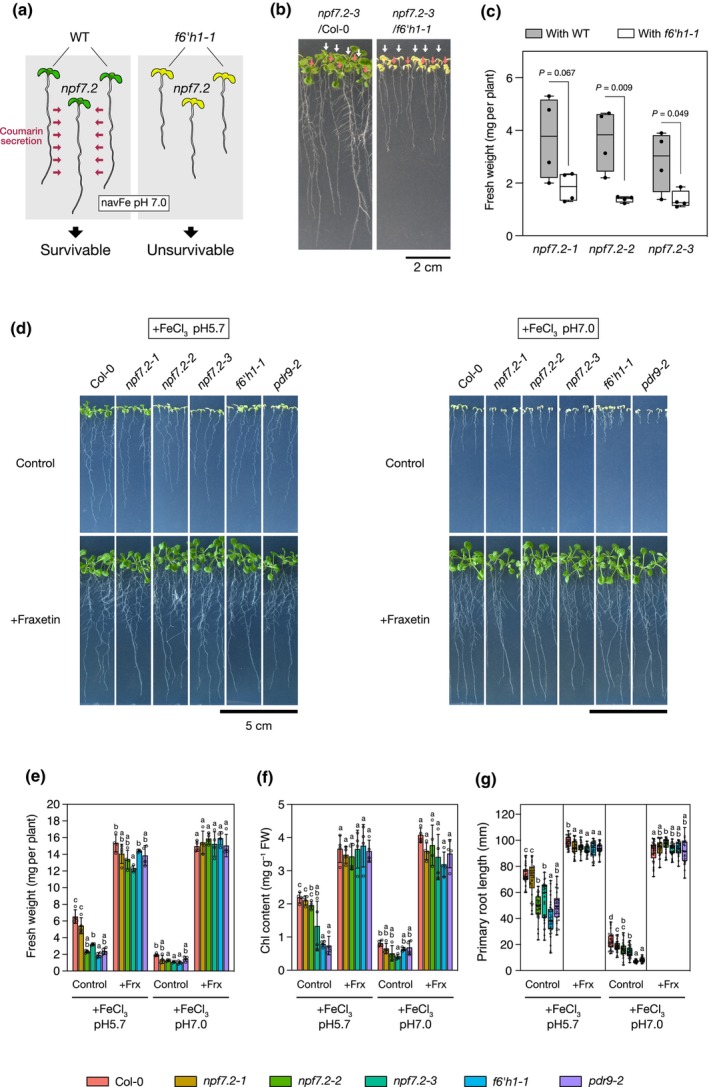
Fraxetin complementation analysis of *npf7.2* mutants. (a) Schematic representation of the experiment. (b) Phenotype of 2‐wk‐old *npf7.2–3* mutant (orange arrows) co‐cultivated with wild‐type (Columbia‐0 (Col‐0)) or *f6′h1‐1* (white arrows) in presence of poorly available iron (Fe) (100 μM FeCl_3_; pH 7.0). Bar, 2 cm. (c) Fresh weight (FW) of *npf7.2* mutants seedlings grown as in (b). Significant differences were detected within each genotype by Student's *t*‐test (*n* = 4). (d) Phenotypes of Col‐0, three *npf7.2* mutant alleles, *f6′h1‐1* and *pdr9‐2* grown for 1 wk in presence of poorly available Fe (100 μM FeCl_3_) supplemented or not with 100 μM of fraxetin. (e–g) FW (*n* = 4) (e), Chl content (*n* = 4) (f) and primary root length (*n* = 28–32) (g) of seedlings described in (a), respectively. Means within each condition with the same letter are not significantly different according to one‐way ANOVA followed by *post hoc* Tukey test, *P* < 0.05. (c, g) Horizontal bars represent the median, boxes represent the middle 50% of the distribution, whiskers represent the entire spread of the data, and dots represent individual measurements. (e, f) Error bars show ± SD. Dots represent individual measurements. *NPF7.2*, NRT1/PTR FAMILY 7.2; NPF, NITRATE TRANSPORTER1/PEPTIDE TRANSPORTER FAMILY.

*f6′h1* mutant's hypersensitivity phenotype to Fe deficiency is due to their inability to synthesize and therefore secrete FMCs into the rhizosphere. Nonetheless, growing *f6′h1* mutants together with WT plants or in the presence of synthetic FMCs (i.e. fraxetin) in the culture medium is sufficient to rescue their Fe‐deficiency‐dependent growth defects (Rodríguez‐Celma *et al*., 2013; Tsai *et al*., 2018; Vanholme *et al*., 2019; Figs 2, S6). Therefore, we investigated whether similar treatments would rescue *npf7.2* mutants' Fe‐deficiency hypersensitive phenotype. We first grew *npf7.2‐3* mutant together with either WT or *f6′h1‐1* seedlings in the presence of poorly available Fe (Fig. 2a,b). We found that co‐cultivation with the WT was sufficient to rescue *npf7.2‐3* Fe‐deficiency growth defects, whereas this was not the case when co‐cultivated with the *f6′h1‐1* mutant (Fig. 2b,c). We then investigated the effect of adding fraxetin into the growth medium. Similar to that observed in *f6′h1‐1*, the Fe‐deficiency growth defect observed in the presence of nonavailable Fe was rescued (including a decrease in FW, Chl content and primary root growth) for the *npf7.2* mutant lines as well as for *pdr9‐2* at both acidic and alkaline pH in a dose‐dependent manner (Figs 2d–g, S6). It is noteworthy that the complementation effect of fraxetin was stronger for *npf7.2‐3* than for *npf7.2‐4*, indicating that *npf7.2‐3* is an hypomorphic allele whereas *npf7.2‐4* is a strong one. Taken together, these data support that *npf7.2* Fe‐deficiency hypersensitivity may be due to a decreased secretion of FMCs as is the case for the *f6′h1‐1* and *pdr9‐2* mutants.

### NPF7.2 expression is induced in peripheral cells of roots in response to Fe deficiency

**Fig. 3:**
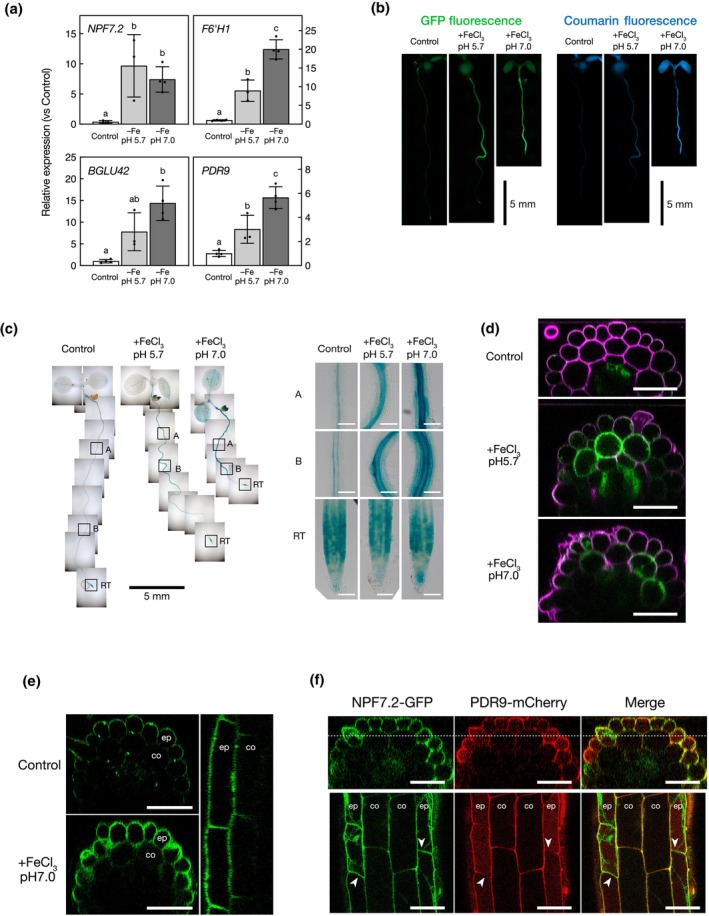
NRT1/PTR FAMILY 7.2 (*NPF7.2*) expression and localization of the encoded protein. (a) Relative expression of *NPF7.2*, FERULOYL‐CoA 6′‐HYDROXYLASE 1 (*F6′H1*), *BGLU42* and *PDR9*. Relative expression was determined by RT‐qPCR in 10‐d‐old Arabidopsis wild‐type (Columbia‐0 (Col‐0)) seedlings grown on iron (Fe)‐sufficient (50 μM Fe‐EDTA) conditions, followed by 3 d of Fe‐deficient treatments. Target gene expression was normalized by *PROTEIN PHOSPHATASE2A SUBUNIT A3*. Means within each condition with the same letter are not significantly different according to one‐way ANOVA followed by *post hoc* Tukey test, *P* < 0.05 (*n* = 3–4). Error bars show ± SD. NPF7.2 mRNA expression was quantified using NPF7.2‐QF2 and NPF7.2‐QR1 primers (Supporting Information Table S1). (b) Left panels: *NPF7.2* promoter activity as revealed by GFP fluorescence in seedlings grown on poorly available Fe (100 μM FeCl_3_). Right panels: Coumarin fluorescence in seedlings displayed in the left panels. Bars, 5 mm. (c) GUS activity driven by the promoter of *NPF7.2* in 10‐d‐old seedlings grown as in (b). Left panels: whole seedlings. Bar, 5 mm. Right panels: zoom within the roots. Bars, 100 μm. (d) GFP fluorescence driven by the promoter of NPF7.2 in the root (at differentiation zone, panels B shown in (c)) of 10‐d‐old seedlings grown as in (b). Bars, 50 μm. (e) NPF7.2‐GFP localization in the roots (at differentiation zone, panels B shown in (c)) of a 10‐d‐old complemented *npf7.2‐3* line (*NPF7.2‐GFP/npf7.2‐3*) grown as in (b). Bars, 50 μm. co, cortex; ep, epidermis. (f) Colocalization of NPF7.2‐GFP and PDR9‐mCherry proteins in the roots at differentiation zone (panels B in (c)) of 9‐d‐old seedlings. Seedlings were grown for 5 d on Fe‐sufficient media and then transferred for 4 d on media deprived of Fe. Upper panels: cross sections. Bars, 50 μm. Lower panels: Longitudinal section on dashed lines in the upper panels. Bars, 50 μm. Arrows indicate the position of NPF7.2‐GFP localization where PDR9‐mCherry accumulation was not detected. co, cortex; ep, epidermis. NPF, NITRATE TRANSPORTER1/PEPTIDE TRANSPORTER FAMILY; EDTA, ethylenediaminetetraacetic acid; *GFP*, *GREEN FLUORESCENT PROTEIN*.

RT‐qPCR analysis confirmed that *NPF7.2* expression was induced in response to Fe deficiency at both acidic and alkaline pH, in which the expression of genes involved in FMCs biosynthesis (i.e. *F6′H1*) and secretion (i.e. *BGLU42* and *PDR9*) was also induced (Fig. 3a; Fourcroy *et al*., 2014; Gao *et al*., 2020). To further analyse the expression of *NPF7.2*, we cloned its promoter fused to the GFP and the GUS reporter genes (*ProNPF7.2:GFP‐GUS*). We found that GFP fluorescence was induced in roots of plants grown under Fe deficiency at both acidic and alkaline pH, in tissues where Fe‐dependent coumarin fluorescence was observed (Fig. 3b). GUS analysis confirmed the induction of *NPF7.2* expression in roots in response to Fe deficiency, except at the root tip where it remains unaffected (Fig. 3c). *ProNPF7.2:GFP‐GUS* analysis also showed that in control condition, *ProNPF7.2* activity was restricted to the root vasculature, whereas under Fe deficiency, it extended to the peripheral tissues (i.e. cortex and epidermis; Fig. 3c,d). To investigate whether NPF7.2 protein accumulation and/or localization is affected by Fe availability, complemented *npf7.2‐3* lines carrying the *ProNPF7.2:gNPF7.2‐GFP* transgene were analysed. We observed slight GFP fluorescence in the vascular zone when seedlings were grown in the control condition (Fig. S7). By contrast, under Fe deficiency, GFP fluorescence was increased drastically at the plasma membrane of the epidermal cells and modestly of the cortical cells (Figs 3e, S8). Colocalization analysis of *ProNPF7.2:gNPF7.2‐GFP* with *ProPDR9:gPDR9‐mCherry* demonstrated that NPF7.2 protein accumulation occurred simultaneously in peripheral cells, in which PDR9 protein was also strongly accumulated, in roots exposed to Fe‐deficient conditions (Fig. 3f). In these cells (i.e. cortex and epidermis), NPF7.2 protein localized all around the plasma membrane, in a nonpolar manner. This is in contrast to PDR9 whose accumulation was shown to be polar, specifically oriented towards the rhizosphere (Ruzicka *et al*., 2010; Robe *et al*., 2021a).

Altogether, these data indicate that Fe deficiency induces *NPF7.2* expression, and the localization of the encoded protein at the plasma membrane of cells that accumulate and secrete FMCs (i.e. cortex and epidermis).

### NPF7.2 loss‐of‐function disrupts Fe‐deficiency‐induced coumarin distribution within roots and secretion into rhizosphere

**Fig. 4:**
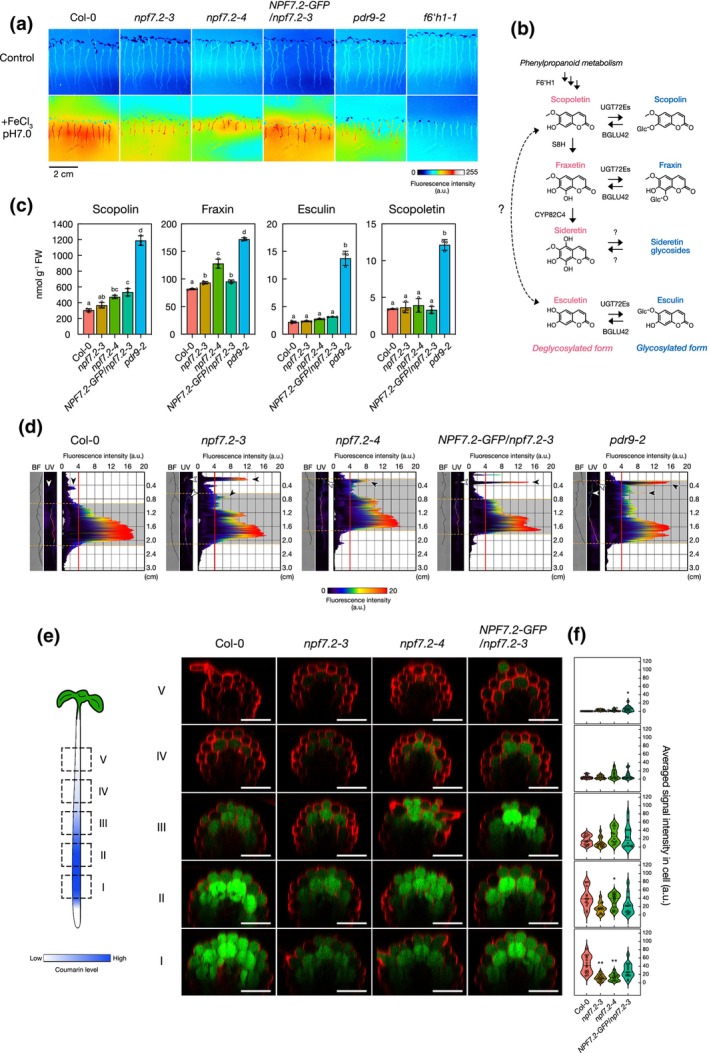
Coumarin accumulation and distribution in *npf7.2* mutants. (a) Coumarin secretion images of Arabidopsis wild‐type (Columbia‐0 (Col‐0)), *npf7.2‐3*, *npf7.2‐4*, the complemented *npf7.2‐3* line (*NPF7.2‐GFP/npf7.2‐3*), *pdr9‐2* and *f6′h1‐1* mutants grown for 1 wk on iron (Fe)‐sufficient (50 μM Fe‐EDTA) conditions or in presence of poorly available Fe (100 μM FeCl_3_; pH 7.0) and then observed under UV light (365 nm). Bar, 2 cm. (b) A representative model of Fe‐mobilizing coumarin (FMC) biosynthesis in Arabidopsis. Enzyme names are written in capital letters. BGLU42, BETA GLUCOSIDASE 42; COSY, COUMARIN SYNTHASE; CYP82C4, CYTOCHROME P450, FAMILY 82, SUBFAMILY C, POLYPEPTIDE 4; F6′H1, FERULOYL‐CoA 6′‐HYDROXYLASE 1; S8H, SCOPOLETIN 8‐HYDROXYLASE; UGT72Es: UDP‐GLUCOSYL TRANSFERASE 72E1 to E3. A dashed double‐headed arrow with question mark symbols indicates that the biochemical steps leading to esculetin biosynthesis from scopoletin have not yet been described. (c) Scopoletin, scopolin (scopoletin glycoside), fraxin (fraxetin glycoside) and esculin (esculetin glycoside) contents in roots of 9‐d‐old seedlings of Col‐0, *npf7.2‐3*, *npf7.2‐4*, *NPF7.2‐GFP/npf7.2‐3* and *pdr9‐2*. Five‐day‐old seedlings grown on Fe‐sufficient media were transferred for 4 d on Fe‐limiting media containing poorly available Fe (100 μM FeCl_3_; pH 7.0). Means within each condition with the same letter are not significantly different according to one‐way ANOVA followed by *post hoc* Tukey test, *P* < 0.05 (*n* = 4). Error bars show ± SD. Dots represent individual measurements. (d) Longitudinal distribution of coumarins in roots of 9‐d‐old seedlings grown as in (c). Grey backgrounds between orange dashed lines on right panels represent areas whose intensities are above the criteria indicated by red lines (> 4 a.u.). White and black arrowheads represent secondary roots and fluorescence intensities corresponding to each secondary root, respectively. BF, root visualization under bright field; UV, coumarin fluorescence in roots upon UV light illumination; cm, distance from the hypocotyl–root transition. (e) Fraxin distribution within roots of 9‐d‐old seedlings after 4 d of Fe‐limiting treatment with poorly available Fe (100 μM FeCl_3_; pH 7.0). Left: model representing site‐specific accumulation of coumarins in roots under Fe‐deficient conditions. Right: representative images of fraxin accumulation within different root zones indicated in the left panel of Col‐0, *npf7.2‐3*, *npf7.2‐4* and complemented *npf7.2‐3* line (*NPF7.2‐GFP/npf7.2‐3*) grown as in (d). Bars, 50 μm. (f) Quantification of cellular fraxin levels in roots of seedlings described in (e). The signal intensity of fraxin was measured in each cell and averaged. Horizontal internal lines indicate the median, and dots represent individual measurements. Asterisks indicate significant differences from Col‐0 in each group detected by Dunn's multiple comparisons test (*, *P* < 0.05; **, *P* < 0.0001, *n* = 12–16). *NPF7.2*, NRT1/PTR FAMILY 7.2; NPF, NITRATE TRANSPORTER1/PEPTIDE TRANSPORTER FAMILY; EDTA, ethylenediaminetetraacetic acid; *GFP*, *GREEN FLUORESCENT PROTEIN*.

To determine whether coumarin secretion was affected in *npf7.2* mutants, coumarin‐derived fluorescence upon UV light exposure was observed in both roots and growth media of seedlings grown on agar plates (Fig. 4a). We detected no obvious difference in the fluorescence emitted from the roots and the growth media of all tested lines grown in control conditions. When grown in presence of poorly available Fe at pH 7, fluorescence signals were increased in the root and diffused in the growth media of WT plants. By contrast, fluorescence in the media around the roots of *npf7.2‐3*, *npf7.2‐4* and *pdr9‐2* mutants was severely reduced under the same conditions, although fluorescence induction was observed in the root of these mutants. The coumarin secretion disruption of *npf7.2‐3* mutant was rescued in the complemented *npf7.2‐3* line by *ProNPF7.2:gNPF7.2‐GFP*. For *f6′h1‐1*, fluorescence levels remained at background level.

To better evaluate the accumulation of coumarins in roots of WT, *npf7.2* mutants, the complemented *npf7.2*, *f6′h1‐1* and *pdr9‐2* seedlings grown in control and poorly Fe available conditions, we quantified scopoletin and glycoside coumarins, scopolin, fraxin and esculin (the main coumarin forms accumulating in Arabidopsis roots) by HPLC (Fig. 4b,c). As reported in a previous report (Robe *et al*., 2021a), these compounds were more highly accumulated in *pdr9‐2* than the WT roots under Fe deficiency. Unlike in *pdr9‐2*, however, the levels of scopolin and fraxin, which are major storage forms in roots, were only slightly higher in *npf7.2* mutants than those in the WT when exposed to Fe deficiency.

*NPF7.2* transcriptional induction in response to Fe deficiency occurred in specific tissues in which coumarins were dominantly accumulated (Fig. 3b). Interestingly, coumarin secretion also initiated in a specific area, which is well in line with the pattern of NPF7.2 protein localization and coumarin accumulation (Fig. S9).

We further analysed coumarin accumulation levels and distribution in *npf7.2* mutant roots to better understand the function of NPF7.2‐mediated coumarin transport on coumarin secretion (Fig. 4d). Coumarin‐derived fluorescence analysis of primary roots showed that even if the intensity of the signal appeared lowered in *npf7.2* mutants when compared to the WT, it was more widely diffused alongside its axis. Interestingly, *npf7.2* mutant displayed also stronger fluorescence signal in secondary roots than the WT. Such patterns of coumarin‐derived fluorescence are consistent with the higher level of coumarin accumulation measured in *npf7.2* mutant when compared to the WT. It is noteworthy that the patterns of coumarin accumulation in *npf7.2* mutants were quite similar to that of *pdr9‐2* even though, fluorescence intensity was lower in *npf7.2* mutants than in *pdr9‐2*, which is also consistent with coumarin quantification. In the complemented *npf7.2‐3* line, coumarin was interestingly still accumulated in the secondary roots, while the broader coumarin distribution in the primary roots was restored to WT levels. This unexpected coumarin distribution might explain the higher quantities of scopolin and fraxin in the whole roots of the complemented *npf7.2‐3* line than those of the WT (Fig. 4c,d).

To determine which coumarin types accumulate alongside the primary root under Fe deficiency, bi‐photon imaging followed by spectral deconvolution was used (Robe *et al*., 2021b; Fig. 4e,f). Of the coumarins that we could visualize, fraxin accumulation was detected in most endodermal, cortical and epidermal cells, in particular, at the Zones I and II of the WT primary roots. In the Zones III, IV and V, fraxin accumulation was drastically decreasing as it neared the hypocotyl–root junction. When compared to the WT, fraxin levels in cortical and epidermal cells were reduced, and fraxin accumulation was not observed in some epidermal cells in Zones I and II of *npf7.2* mutant roots. This disrupted accumulation in *npf7.2‐3* was complemented at least partially by the introduction of *ProNPF7.2:gNPF7.2‐GFP*. Besides, we analysed scopolin accumulation and distribution and found no obvious difference between the WT and *npf7.2* mutants (Fig. S10).

### NPF7.2 has an uptake activity for iron mobilizing coumarin

**Fig. 5:**
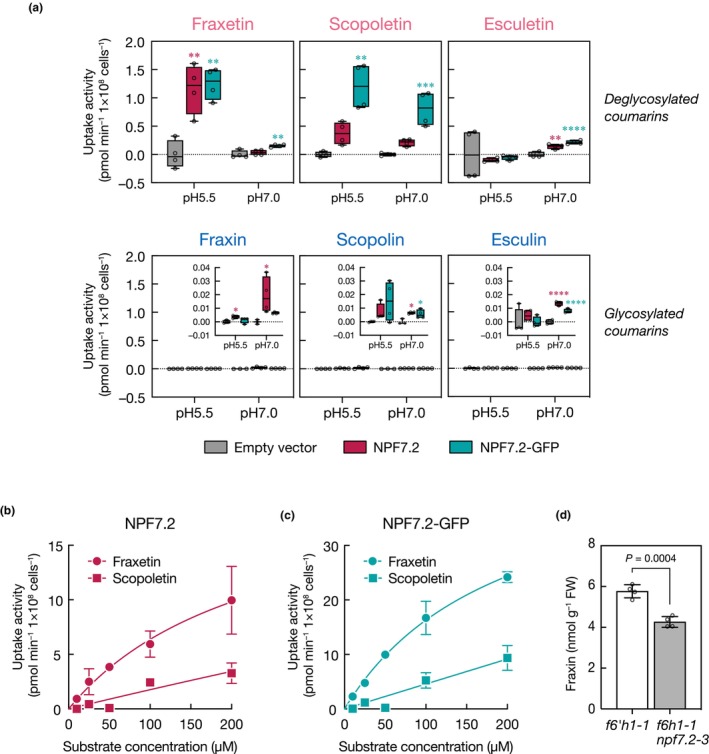
Coumarin uptake activity of NRT1/PTR FAMILY 7.2 (NPF7.2). (a) Heterologous transport assay of NPF7.2 and NPF7.2‐GFP proteins against different coumarin compounds using yeast cells at pH 5.5 and pH 7.0 conditions. Activities were determined in the presence of each substrate at a 50 μM concentration. Yeast cells transformed with the empty vector (pAG426GAL) were used as a control. Boxes represent the middle 50% of the distribution, and whiskers represent the entire spread of the data (*, *P* < 0.05; **, *P* < 0.01; ***, *P* < 0.001 against an empty vector control by Tukey's multiple comparison test, *n* = 4). Dots represent individual measurements. Insets show the enlarged views of the transport activities in −0.01 to 0.04 pmol min^−1^ 1 × 10^8^ cells^−1^. (b, c) Uptake transport kinetics of NPF7.2 and NPF7.2‐GFP proteins against fraxetin and scopoletin, which are the major iron (Fe)‐mobilizing coumarins (FMC) and its precursor, respectively, at pH 5.5. The amount of the compounds taken into the yeast cells expressing NPF7.2 was determined when the substrate concentrations were 10, 25, 50, 100 and 200 μM. Data are shown as means of three or four independent biological repeats. Solid lines indicate nonlinear curve fits of the data to the Michaelis–Menten equation. Error bars show ± SD. (d) Fraxin levels of *f6′h1‐1* and *f6′h1‐1 npf7.2‐3* double mutant roots when treated with 20 μM fraxetin. Data are shown as mean ± SD. A significant difference was determined by Student's *t*‐test (*n* = 4). NPF, NITRATE TRANSPORTER1/PEPTIDE TRANSPORTER FAMILY; *GFP*, *GREEN FLUORESCENT PROTEIN*.

To determine whether NPF7.2 protein has transport activity for coumarin compounds, a direct transport assay was performed using heterologous expression of NPF7.2 and NPF7.2‐GFP fusion proteins in yeast cells. Each coumarin compound incorporated into yeast cells heterologously expressing NPF7.2 or NPF7.2‐GFP proteins was quantified using HPLC after incubating the cells with the candidate transport substrates. We observed an uptake activity of NPF7.2 and NPF7.2‐GFP proteins against fraxetin and scopoletin when the reaction buffer was weakly acidic (pH 5.5; Fig. 5a). However, esculetin was not shown to be incorporated into cells expressing either NPF7.2 or NPF7.2‐GFP under the same conditions. Their corresponding glycosides, fraxin, scopolin and esculin were also tested to evaluate whether they could be a transport substrate of NPF7.2. A small amount of the glycosides was taken into NPF7.2‐ or NPF7.2‐GFP‐expressing cells; however, their uptake velocities were quite low (between 0 and 0.04 pmol min^−1^ 1 × 10^8^ cells), unlike the aglycones. Since NPF members function as proton‐coupled transporters, we further assessed the transport activity against these compounds at pH 7.0. As expected, the uptake activity for fraxetin was diminished by equivalating the proton gradient between the inner and outer cells. Interestingly, scopoletin uptake activity in the same pH environment was slightly affected. Kinetic analysis using Michaelis–Menten plots indicated that the *K*_m_ values for NPF7.2 and NPF7.2‐GFP proteins regarding fraxetin were 233.7 and 196.2 μM, and *V*_max_ values were 22.16 and 48.26 pmol/min/1 × 10^8^ cells, respectively (Fig. 5b,c). On the contrary, the kinetics of scopoletin uptake showed a dose‐dependent response between 0 and 200 μM of the substrate and it did not follow the Mikaelis–Menten equation, which prevented calculating *K*_m_ and *V*_max_ values.

Since NPF7.2 mediates cellular coumarin uptake when expressed in yeast, we speculated that NPF7.2 is involved not only in coumarin secretion but also in coumarin uptake from rhizosphere into the root tissues. To address this possibility, we quantified level of fraxin, a storage form of fraxetin, in roots of *f6h1‐1 npf7.2‐3* double knock‐out mutant grown in presence of fraxetin in the media and compared it to the one of *f6h1‐1* (Fig. 5d). A lower accumulation of fraxin was observed in *f6h1‐1 npf7.2‐3* when compared to *f6h1‐1*, providing evidence that NPF7.2 functions as an import transporter of coumarins in roots.

## Discussion

Genome‐wide gene expression analysis using Arabidopsis roots exposed to Fe‐deficient stress allowed us to select NPF7.2 as a potential transporter of coumarins (Fig. S1). While NPF7.2 was described as a nitrate transporter related to long‐distance allocation of nitrate (Li *et al*., 2010; Wang *et al*., 2012), its expression is induced in response to Fe deficiency (Colangelo & Guerinot, 2004) and is tightly correlated with that of *BGLU42* (Zamioudis *et al*., 2014).

The phenotypic analyses of the mutants performed in this work demonstrated that NPF7.2 loss‐of‐function inhibited seedling growth in the presence of FeCl_3_, which is a poorly available form of Fe for plant roots, as the sole Fe source in the media at both acidic and alkaline pH (Figs 1, S2d–g). These phenomena are typical of plants suffering from Fe deficiency, and similar phenotypes to those of *npf7.2* mutants were observed in mutants defective in coumarin biosynthesis and secretion, such as *f6′h1‐1* and *pdr9‐2*, respectively (Fig. S2d–g; Rodríguez‐Celma *et al*., 2013). In fact, endogenous Fe levels in *npf7.2‐3* and *npf7.2‐4* mutants were significantly less than that of the WT (Fig. S4). The growth retardations of *npf7.2* seedlings were rescued by exogenously supplying fraxetin (Figs 2d–g, S6). In addition, the co‐cultivation of WT plants capable of secreting coumarins from roots exerted the same effects; meanwhile, no recovery effect was observed when co‐cultivating with *f6′h1‐1* (Fig. 2a–c). Taking into account the poor coumarin secretion of *npf7.2* mutants (Fig. 4a), these results suggest that NPF7.2 contributes to root Fe acquisition in Arabidopsis under low bioavailable Fe conditions by facilitating coumarin secretion.

pH around the roots is a factor affecting Fe bioavailability for plants and thus the gene regulatory network controlling Fe acquisition and homeostasis (Tsai & Schmidt, 2020; Gautam *et al*., 2021; Hartemink & Barrow, 2023). As a response, coumarin exudation composition is finely tuned according to soil pH (Rajniak *et al*., 2018). For instance, CYP82C4, which catalyses the fraxetin‐to‐sideretin conversion, is upregulated at acidic pH, leading to the secretion of sideretin (Fig. 4b). At the transcriptional level, no difference in *NPF7.2* expression was observed between acidic and alkaline pH conditions (Fig. 3a). Accordingly, *npf7.2* mutants displayed pH‐independent phenotypes, although the growth inhibition was more severe under alkaline than under acidic condition (Fig. 1). Similar trends were noted for F6′H1 and PDR9, which play an essential role in coumarin biosynthesis and secretion across a broad range of pH, supporting the role of NPF7.2 in coumarin secretion‐based Fe acquisition, independently of the surrounding pH. It also suggests that NPF7.2 may also mediate cellular transport of sideretin as for fraxetin.

Under Fe deficiency, similarly to PDR9, NPF7.2 accumulates in root cortical and epidermal cells, which are the major sources of FMCs for secretion (Figs 3b–f, 4d–f, S8, S10; Schmid *et al*., 2014; Rajniak *et al*., 2018; Robe *et al*., 2021a). In the absence of NPF7.2, Fe‐deficiency‐induced coumarin secretion was negatively affected (Fig. 4a), supporting the involvement of NPF7.2 in this process. NPF7.2 was also detected in vascular tissues, in agreement with previous studies, where NPF7.2 was associated with nitrate retrieval from xylem (Figs 3c,d, S7, S8; Li *et al*., 2010). The vasculature‐specific accumulation of NPF7.2 protein presented here did not appear to be affected by the Fe deficiency (Fig. 3e,f), except for its accumulation probably in endoplasmic reticulum in vascular tissues during the early stages of Fe deficiency, such as within 3 d of treatment (Fig. S8). This may reflect a strong initial induction of NPF7.2 expression before post‐translational regulation of its plasma membrane localization. These spatiotemporal differences of expression patterns likely indicate that the two distinct functions of NPF7.2, as a nitrate and coumarin transporter, may be regulated independently of each other. It is worth mentioning that NPF7.2 is evenly distributed on the plasma membrane whereas PDR9 is polarly localized at the outermost plasma membrane in peripheral cells (Fig. 3e,f; Robe *et al*., 2021a). The ubiquitous distribution of NPF7.2 on the plasma membrane might also explain the multiple functions of the transporter.

**Fig. 6:**
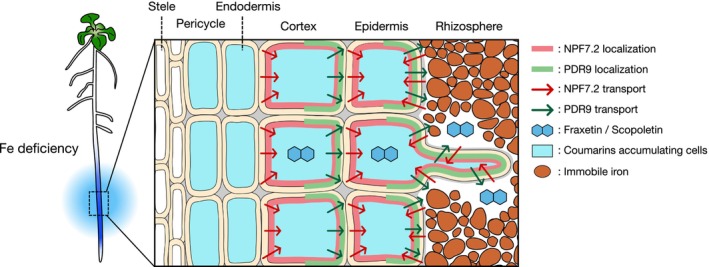
Model of the site‐specific coumarin secretion coordinately driven by NRT1/PTR FAMILY 7.2 (NPF7.2) and PDR9. In response to iron (Fe) deficiency, intensive accumulation and secretion of coumarins occur in the specific area of Arabidopsis roots (highlighted with blue in the left diagram). NPF7.2 accumulation is strongly induced in cortical and epidermal cells of this region, where PDR9 also accumulates simultaneously. In this area, NPF7.2 mediates the cellular loading of coumarins into peripheral tissues, facilitating PDR9‐mediated Fe‐mobilizing coumarin (FMC) secretion from the epidermis into the rhizosphere. Thick pink and pale green lines indicate the localization of NPF7.2 and PDR9 proteins, respectively. Red and green arrows represent the influx and efflux flows of coumarin compounds mediated by NPF7.2 and PDR9, respectively. NPF, NITRATE TRANSPORTER1/PEPTIDE TRANSPORTER FAMILY.

We observed that root coumarin accumulation in *npf7.2* mutants was not as pronounced as in *pdr9‐2*, despite the severe inhibition of coumarin secretion (Fig. 4a,c). Moreover, when compared at tissue and cellular levels, coumarins are less accumulated in *npf7.2* in the specific area of roots even if compared to WT (Fig. 4d–f). One possible explanation for these results would be that NPF7.2 is involved in the uptake of coumarins from the rhizosphere to the root tissues. In fact, fraxin uptake was impaired in the *f6′h1‐1 npf7.2‐3* double mutant (Fig. 5d). We speculate that NPF7.2 functions in concentrating coumarins in root cortical and epidermal cells, and this is probably required for efficient coumarin export from the root tissues mediated by PDR9 (Fig. 6). This idea is supported by the observation that fraxin is less accumulated in the cortex and epidermis of *npf7.2* compared with WT (Fig. 4e,f). Further understanding on feedback/feedforward regulation of coumarin metabolism as well as identification of additional coumarin transporters will be required to fully depict the FMC secretion process.

Direct transport assay revealed that NPF7.2 protein has an uptake activity for fraxetin, and its precursor scopoletin, two major forms of coumarin aglycones in Arabidopsis roots (Figs 4b, 5a). The kinetics of uptake activities against these compounds suggest that fraxetin is a preferred substrate of NPF7.2 than scopoletin (Fig. 5b,c). In contrast to these compounds, no remarkable uptake activity of NPF7.2 was found for esculetin or coumarin glycosides that are the typical cellular storage forms (Fig. 5a).

NPF members are widely recognized as proton‐coupled symporters, and a pH gradient across membranes is required to generate their transport activities (Léran *et al*., 2014; Jørgensen *et al*., 2017). As expected, in our yeast assays, fraxetin uptake activity of NPF7.2 observed at pH 5.5 was diminished at pH 7.0 (Fig. 5a). This observation is in contrast to the fact that fraxetin secretion by the plant root occurs at alkaline pH (Sisó‐Terraza *et al*., 2016). How NPF7.2‐mediated coumarin uptake is stimulated in plants at such pH remains to be elucidated. One possible explanation for this observation is that plasma membrane proton‐ATPases may work in coordination with NPF7.2 to generate local proton gradients by acidifying the rhizosphere. For instance, like *AHA2*, the expression of several *AHA* family members is induced in roots in response to Fe‐deficiency conditions (Santi & Schmidt, 2009; Martín‐Barranco *et al*., 2020). In addition, a recent study reported that NPF7.3, the closest homologue of NPF7.2, physically interact with AHA2, which is required for its K^+^ export activity in plants (Sena & Kunze, 2023).

Contrary to the fraxetin, pH has moderate effect on the uptake activity of NPF7.2 in yeast for scopoletin (Fig. 5a). Unlike most NPF that possess an EXXER/K motif reported to confer proton‐driven symport activity of PROTON‐COUPLED OLIGOPEPTIDE TRANSPORTER superfamily (Daniel *et al*., 2006; Léran *et al*., 2014; Newstead, 2015; Jørgensen *et al*., 2017), NPF7.2 is deprived of it. Interestingly, NPF lacking this motif, such as NAXT and FST1, are suggested to be passive transporters of nitrate and flavonoid glycosides, respectively (Segonzac *et al*., 2007; Grunewald *et al*., 2020), suggesting that NPF7.2 scopoletin uptake activity may be driven in a pH‐independent manner.

## Competing interests

None declared.

## Author contributions

SW, MS and CD conceived and designed the experiments. SW, ML, AR, CB, KR and YK performed the experiments. SW, ML, AR, CB, KR, YK, MS and CD analysed the data. SW, MS and CD contributed reagents/materials/analysis tools. SW and CD wrote the article.

## Disclaimer

The New Phytologist Foundation remains neutral with regard to jurisdictional claims in maps and in any institutional affiliations.

## Supporting information


**Fig. S1** Identification of candidate genes involved in Fe‐mobilizing coumarin transport in Arabidopsis.
**Fig. S2** Phenotypes of *npf7.2* T‐DNA insertion mutants in Fe‐limiting conditions.
**Fig. S3** Generating NPF7.2 gene deletion mutants using CRISPR/Cas9 system.
**Fig. S4** Fe contents in *npf7.2* mutants.
**Fig. S5** NPF7.2 mRNA expression in the complementation line of *npf7.2‐3*, transformed by *proNPF7.2:gNPF7.2‐GFP*.
**Fig. S6** Dose‐dependent complementation of *npf7.2* mutant phenotypes by fraxetin.
**Fig. S7** NPF7.2 protein localization in vasculature tissues of roots under Fe‐sufficient conditions.
**Fig. S8** NPF7.2 protein expression in roots in the absence of Fe at acidic and alkaline pH.
**Fig. S9** Initial sites of coumarin secretion.
**Fig. S10** Scopolin distribution in *npf7.2* mutants roots.
**Table S1** Primers and synthetic DNA used in this study.

**Table S2** mRNA expression of transporter genes in Arabidopsis roots under iron deficiency.
Please note: Wiley is not responsible for the content or functionality of any Supporting Information supplied by the authors. Any queries (other than missing material) should be directed to the *New Phytologist* Central Office.

## Data Availability

The data that support the findings of this study are available in the Figs S1–S10, Tables S1 and S2 of this article.
