# Supplementary material for: The Arabidopsis NPF7.2 mediates coumarin uptake for root iron acquisition
Source: New Phytol. 2026 Mar 2;250(3):1619–33. doi: 10.1111/nph.70993 (PMC13062693; doi:10.1111/nph.70993)
Supplement: Supplementary file 1 — Fig. S1 Identification of candidate genes involved in Fe‐mobilizing coumarin transport in Arabidopsis. Fig. S2 Phenotypes of npf7.2 T‐DNA insertion mutants in Fe‐limiting conditions. Fig. S3 Generating NPF7.2 gene deletion mutants using CRISPR/Cas9 system. Fig. S4 Fe contents in npf7.2 mutants. Fig. S5 NPF7.2 mRNA expression in the complementation line of npf7.2‐3, transformed by proNPF7.2:gNPF7.2‐GFP. Fig. S6 Dose‐dependent complementation of npf7.2 mutant phenotypes by fraxetin. Fig. S7 NPF7.2 protein localization in vasculature tissues of roots under Fe‐sufficient conditions. Fig. S8 NPF7.2 protein expression in roots in the absence of Fe at acidic and alkaline pH. Fig. S9 Initial sites of coumarin secretion. Fig. S10 Scopolin distribution in npf7.2 mutants roots. Table S1 Primers and synthetic DNA used in this study. [file NPH-250-1619-s002.pdf]

New Phytologist

New Phytologist Supporting Information

Article title: The Arabidopsis NPF7.2 mediates coumarin uptake for root iron acquisition
Authors: Shunsuke Watanabe, Meijie Li, Alice Rossille, Chérhazad Boustani, Kevin Robe, Yuri Kanno, Mitsunori Seo and Christian Dubos
Article acceptance date: 14 January 2026

The following Supporting Information is available for this article:

Fig S1

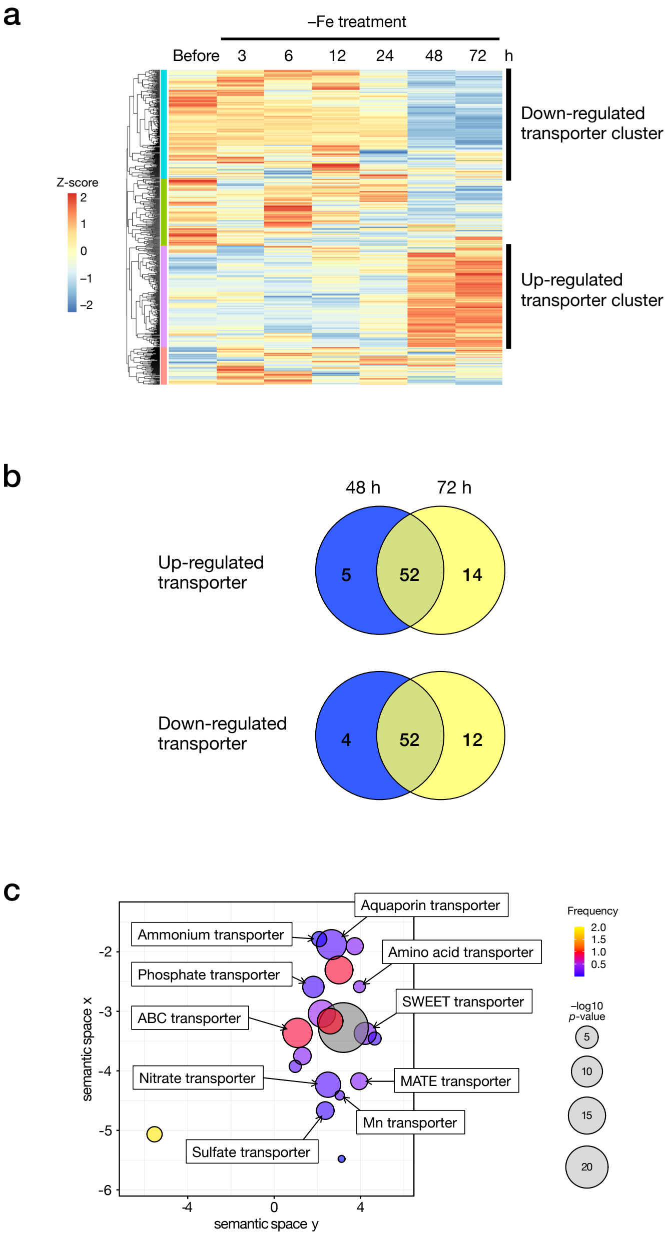

Fig S1. Identification of candidate genes involved in Fe mobilizing coumarin transport in Arabidopsis.

(a) Hierarchical clustering analysis of 668 transporter genes classified as "Transporter activity" in the TAIR database. Transcriptome data from Fe-deficient Arabidopsis roots deposited in the Gene Expression Omnibus [GSE10576; Dinneny *et al.* (2008)) was used. **(b)** Comparison of differentially expressed transporter genes (DEGs) between 48 and 72 hours of Fe-deficiency treatments. DEG whose expression was increased or decreased more than 2-fold was identified in a comparison between Fe-deficiency treatment and before treatment (corrected $P < 0.05$ by one-way ANOVA). **(c)** Gene ontology (GO) classification for molecular function of the upregulated and downregulated transporter genes isolated in (a).

New Phytologist

Fig S2

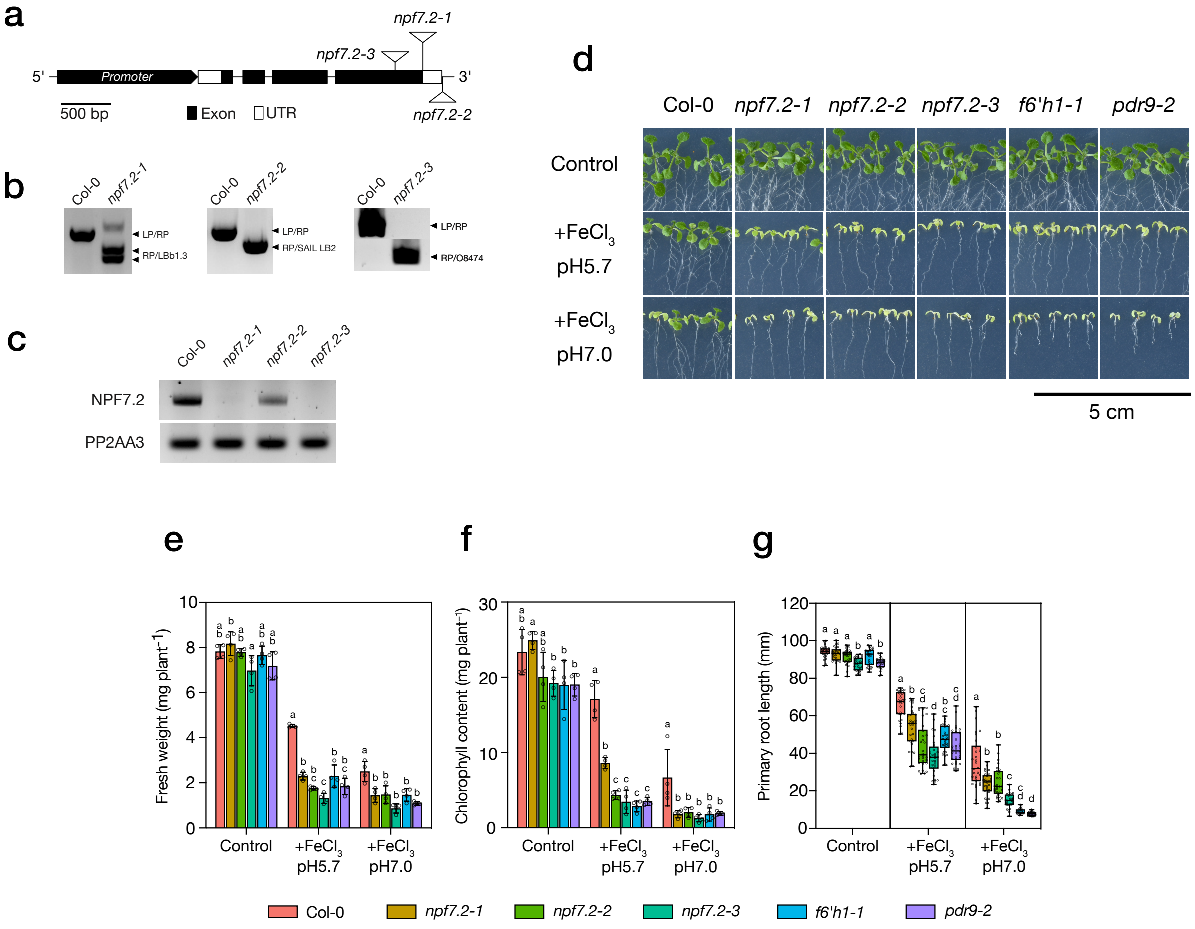

Fig S2. Phenotypes of *npf7.2* T-DNA insertion mutants in Fe-limiting conditions.

(a) Gene structure of *NPF7.2* gene and the location of T-DNA insertion in each mutant. **(b and c)** T-DNA insertion **(b)** and suppressed expression **(c)** of *NPF7.2* gene in *npf7.2* mutants. **(d)** Fe deficiency hyper-sensitive phenotypes of *npf7.2* mutants. Seedlings were grown for one week on Fe-sufficient (50 µM Fe-EDTA) condition or in presence of poorly available Fe (100 µM $FeCl_3$). Bar = 5 cm. **(e-g)** Fresh weight (n = 4) **(e)**, chlorophyll content (n = 4) **(f)** and primary root length (n = 30-41) **(g)** of seedlings described in (d), respectively. Means within each condition with the same letter are not significantly different according to one-way ANOVA followed by post hoc Tukey test, $P < 0.05$. **(e and f)** Error bars show ±SD. Dots represent individual measurements. **(g)** Horizontal bars represent the median, boxes represent the middle 50% of the distribution, whiskers represent the entire spread of the data, and dots represent individual measurements.

Fig S3

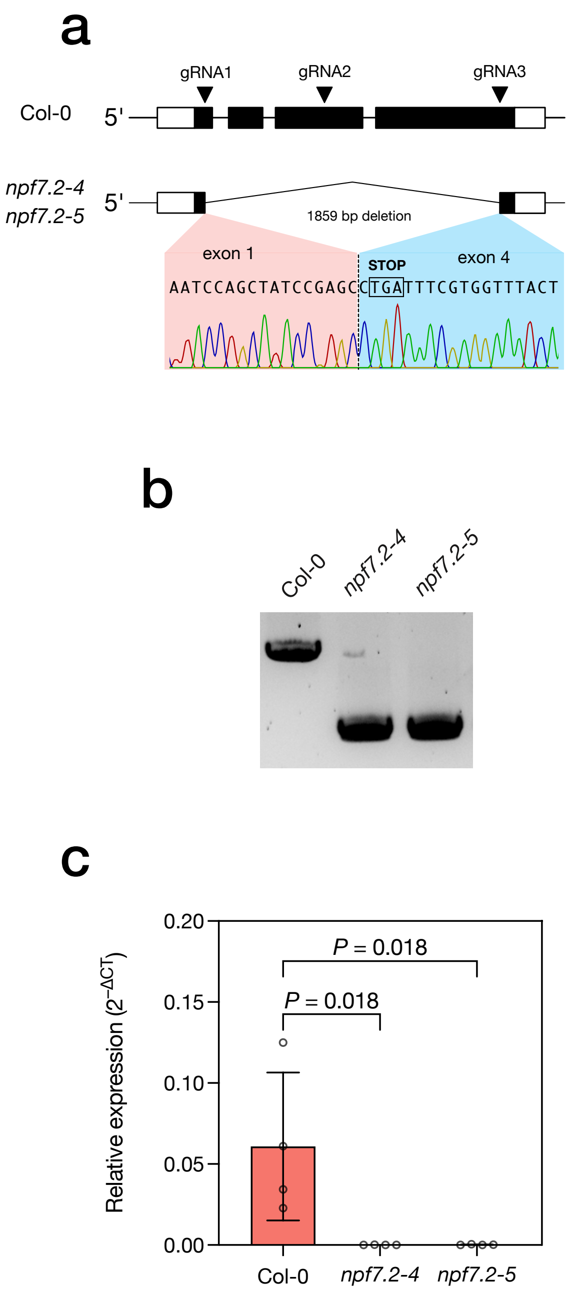

Fig S3. Generating NPF7.2 gene deletion mutants using CRISPR/Cas9 system.

(a) Guide RNA (gRNA)-targeted sites in *NPF7.2* gene and the resulting gene deletion in the mutants. **(b)** PCR for confirmation of the *NPF7.2* deletion in the mutants as shown in (a). **(c)** mRNA expression of NPF7.2 in the mutant as shown in (a). Data are shown as mean ± SD. Dots represent individual measurements. A significant difference was determined by Student's *t*-test (n = 4). NPF7.2 mRNA expression was quantified using NPF7.2-QF2 and NPF7.2-QR1 primers (Supplementary Table S1).

New Phytologist

Fig S4

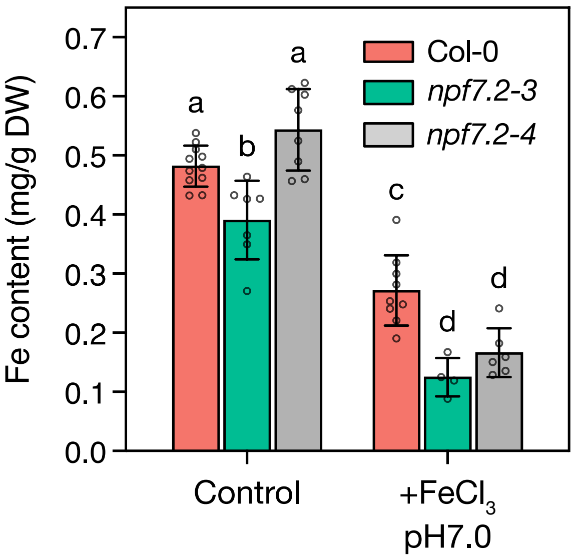

Fig S4. Fe contents in *npf7.2* mutants.

Endogenous Fe levels in wild type, *npf7.2-3* and *npf7.2-4* mutant seedlings. Seedlings were grown for two weeks on Fe-sufficient conditions (50 µM Fe-EDTA) or in presence of poorly available Fe (100 µM $FeCl_3$). Data are shown as mean ± SD. Means with the same letter are not significantly different according to one-way ANOVA followed by post hoc Tukey test, $P < 0.05$ (n = 4-11). Dots represent individual measurements.

Fig S5

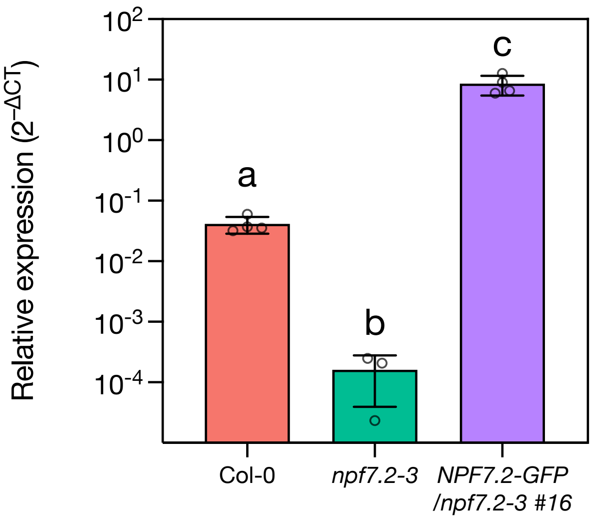

Fig S5. NPF7.2 mRNA expression in the complementation line of *npf7.2-3,* transformed by *proNPF7.2:gNPF7.2-GFP*.

mRNA expression of NPF7.2 in the complementation line. Data are shown as mean ± SD. Means with the same letter are not significantly different according to one-way ANOVA followed by post hoc Tukey test, $P < 0.05$ (n = 3-4). Dots represent individual measurements. NPF7.2 mRNA expression was quantified using NPF7.2-QF3 and NPF7.2-QR2 primers (Supplementary Table S1).

Fig S6

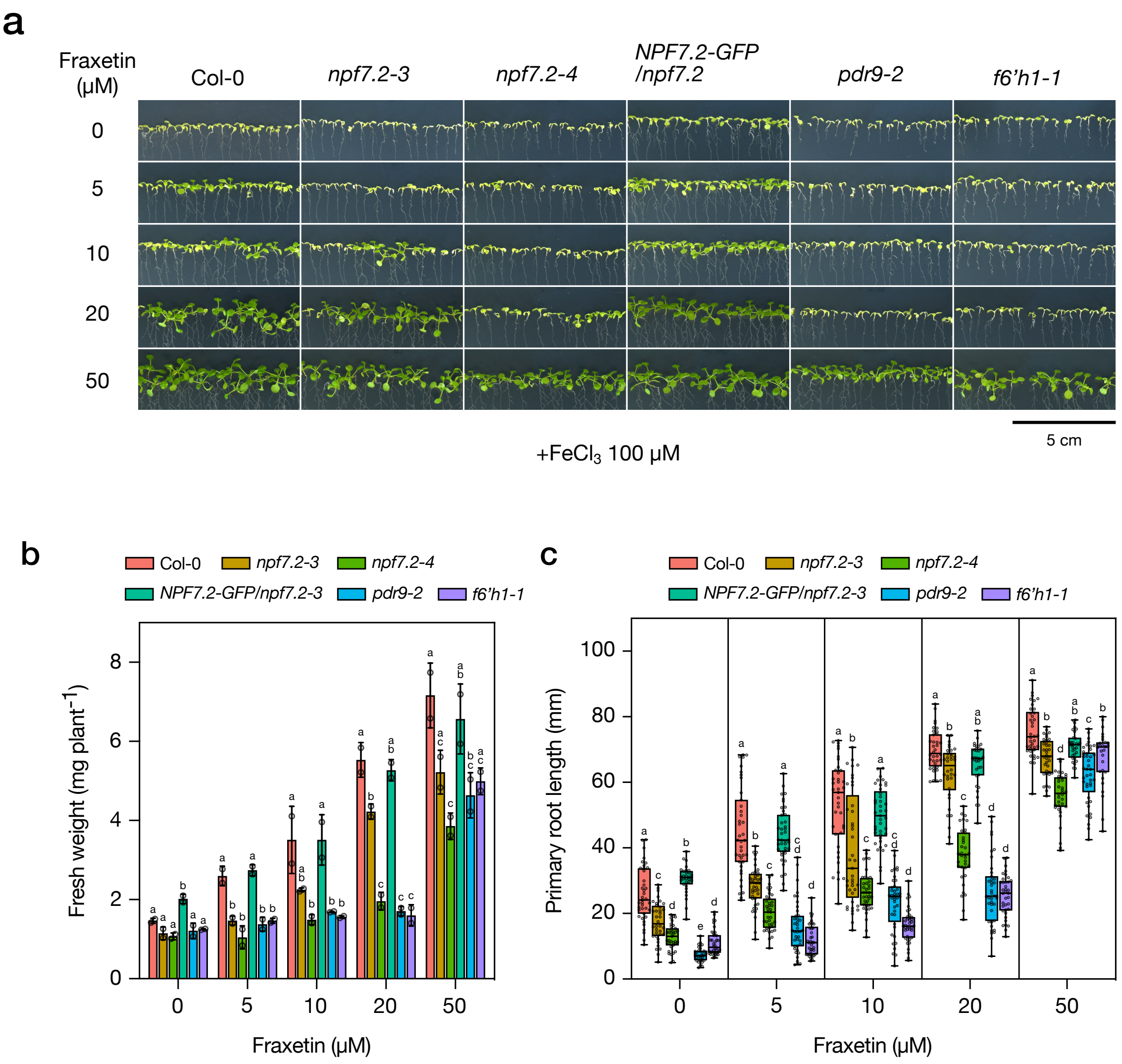

Fig S6. Dose-dependent complementation of *npf7.2* mutant phenotypes by fraxetin.

(a) Representative images of Arabidopsis wild type (Col-0), *npf7.2-3*, *npf7.2-4*, the complemented *npf7.2-3* line (*NPF7.2-GFP/npf7.2-3*), *pdr9-2* and *f6'h1-1* mutants grown for 12 days on poorly available Fe (100 µM FeCl3) media supplemented with fraxetin at the indicated concentrations (0, 5, 10, 20 and 50 µM). Bar = 5 cm. **(b-c)** Fresh weight (n = 2) **(b)** and primary root length (n = 28-41) **(c)** of seedlings described in (a), respectively. Means within each condition with the same letter are not significantly different according to one-way ANOVA followed by post hoc Tukey test, $P < 0.05$. (b) Error bars show ±SD. Dots represent individual measurements. **(c)** Horizontal bars represent the median, boxes represent the middle 50% of the distribution, whiskers represent the entire spread of the data, and dots represent individual measurements.

Fig S7

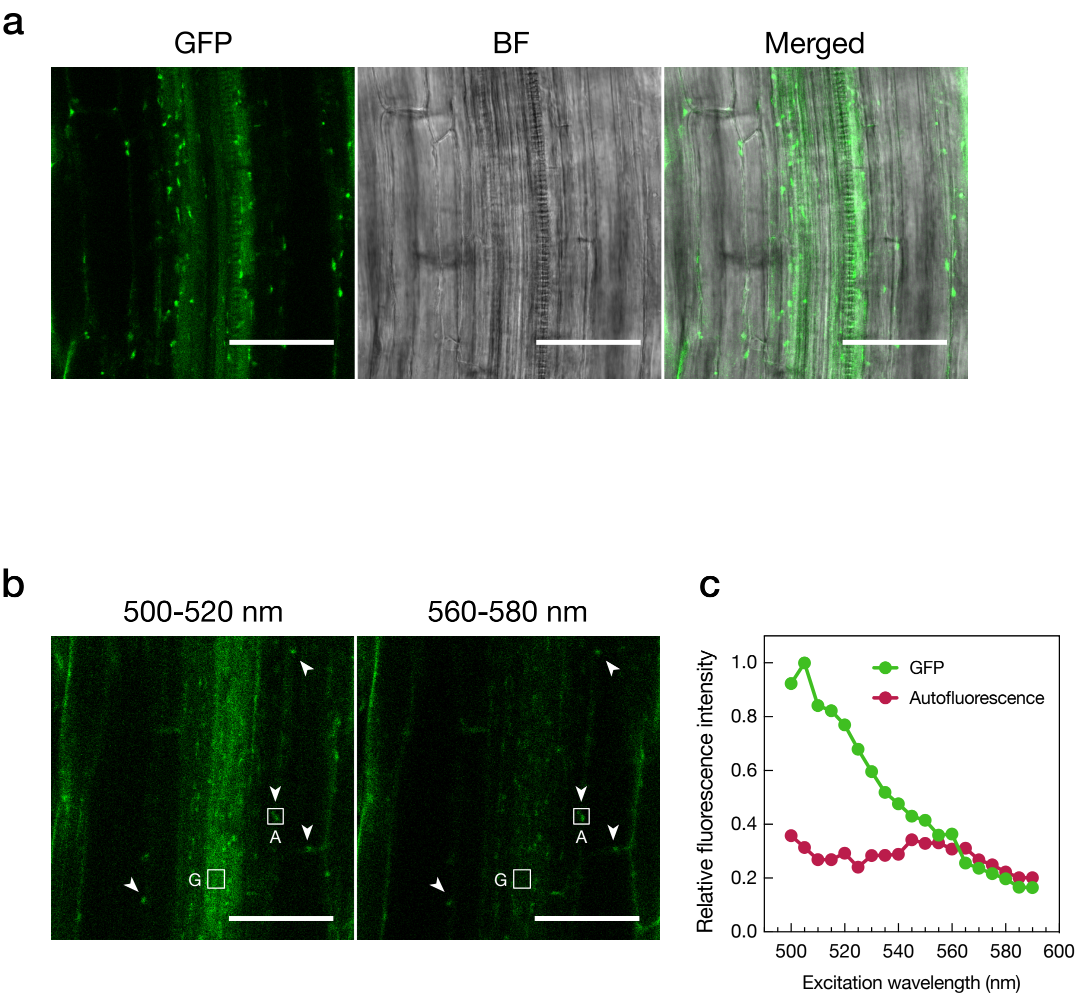

Fig S7. NPF7.2 protein localisation in vasculature tissues of roots under Fe-sufficient conditions.

(a) NPF7.2-GFP fluorescence detected in vasculature tissues of primary roots in 7-day-old seedlings grown under Fe-sufficient conditions (50 µM Fe-EDTA). Bar = 50 µm. **(b)** Different fluorescence spectra emitted from GFP and auto-fluorescent organelles (white arrowheads). Left panel: representative fluorescence images from roots grown as in (a). Right panel: averaged fluorescence spectrum obtained from white frames (G and A in the right panel).

Fig S8

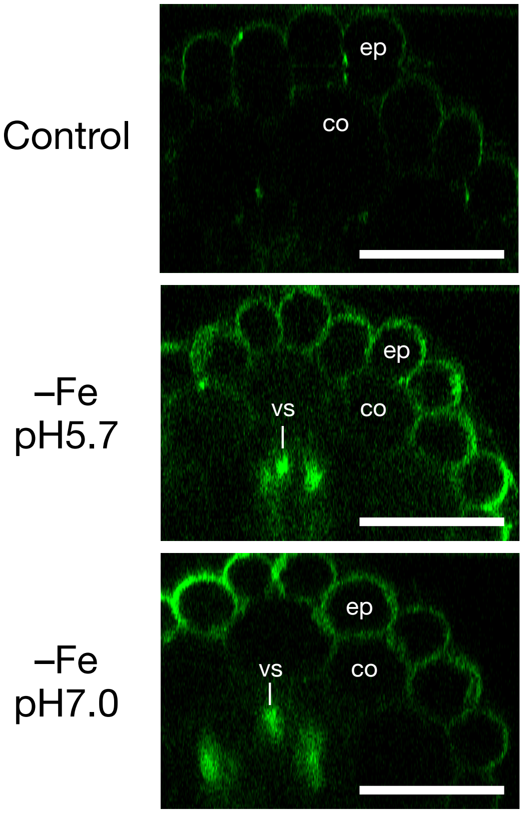

Fig S8. NPF7.2 protein expression in roots in the absence of Fe at acidic and alkaline pH. NPF7.2-GFP localization in the roots [at differentiation zone, panels B shown in (c)] of 9-day-old seedlings of the complemented npf7.2-3 line (NPF7.2-GFP/npf7.2-3). Seedlings were grown for five-day-old on Fe-sufficient media and then transferred for 3 days on media deprived of Fe. Bar = 50 µm. ep: epidermis, co: cortex, pr: pericycle.

Fig S9

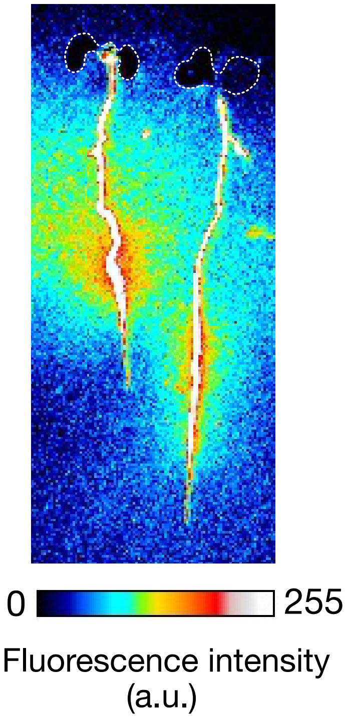

Fig S9. Initial sites of coumarin secretion.

Initial sites of coumarin secretion of the Col-0 roots grown for one week on half-MS media in presence of poorly available Fe (100 µM FeCl3; pH 7.0) and then observed under UV light (365 nm). Bar = 2 cm. Dashed lines delimit shoot parts of the seedlings.

Fig S10

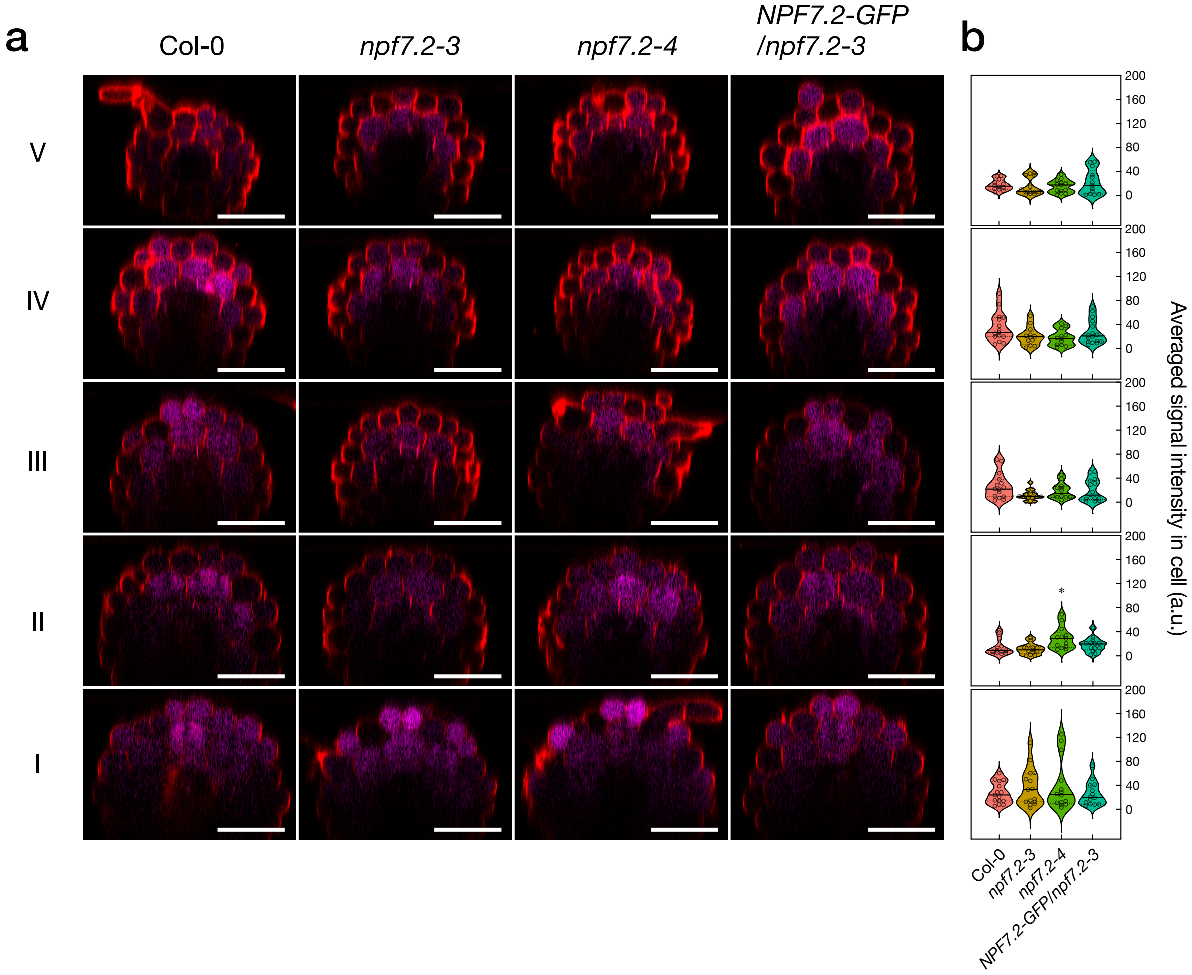

Fig S10. Scopolin distribution in *npf7.2* mutants roots.

(a) Representative images of scopolin (scopoletin glycoside) accumulation within different root zones (indicated in the left panel in Fig. 5f) of 9-day-old seedlings of Arabidopsis wild type (Col-0), *npf7.2-3*, *npf7.2-4* and complemented *npf7.2-3* line (*NPF7.2-GFP/npf7.2-3*) after 4 days of the Fe-limiting treatment with poorly available Fe (100 µM FeCl3; pH 7.0). Bar = 50 µm. **(b)** Quantification of cellular scopolin levels in roots of Col-0, *npf7.2-3*, *npf7.2-4* and complemented *npf7.2-3* line (*NPF7.2-GFP/npf7.2-3*) upon Fe-limiting treatment. The signal intensity of scopolin was measured in each cell and averaged. Horizontal internal lines indicate the median and dots represent individual measurements. Asterisks indicate significant differences from Col-0 in each group detected by Dunn's multiple comparisons test (*$P < 0.05$, n = 12-16).

Supplementary Table S1. Primers and synthetic DNA used in this study.

Target*	Use for	Forward primer		Reverse primer	
		Name	Sequence (5'-3')	Name	Sequence (5'-3')
***npf7.2* mutants generation and isolation**					
NPF7.2/NRT1.8†	PCR genotyping	NPF7.2g-F1	CTCAGTGTCGCCTTCTTCATC	NPF7.2g-R1	GATCAAGACAGCGAGAGTTGC
NPF7.2/NRT1.8†	PCR genotyping	NPF7.2g-F2	GCCATCTTCGGGAAGATACTC	NPF7.2g-R2	GCAACAAAGGTCTCACTGAGC
NPF7.2/NRT1.8†	PCR genotyping	NPF7.2g-F3	GATGTGCACCATGAAGAGTTG	NPF7.2g-R3	TGTTGGAACTGTGGAATCAAC
NPF7.2/NRT1.8	PCR genotyping	NPF7.2g-F4	CAGGTGTAACTGGTCACACATTC	NPF7.2g-R3	TGTTGGAACTGTGGAATCAAC
T-DNA	PCR genotyping	LBb1.3	ATTTTGCCGATTTCGGAAC		
T-DNA	PCR genotyping	O8474	ATAATAACGCTGCGGACATCTACATTTT		
NPF7.2 guide RNA	guide RNA generation	NPF7.2-gRNA1-st	attgGCAGACGCCCAAAATCCAAG	NPF7.2-gRNA1-cp	aaacCTTGGATTTTGGGCGTCTGC
NPF7.2 guide RNA	guide RNA generation	NPF7.2-gRNA2-st	attgGTAAACCACGAAATCAGCAG	NPF7.2-gRNA2-cp	aaacCTGCTGATTTCGTGGTTTAC
NPF7.2 guide RNA	guide RNA generation	NPF7.2-gRNA3-st	attgGCCATTTGCCGGTATTAGCT	NPF7.2-gRNA2-cp	aaacAGCTAATACCGGCAAATGGC
Gateway cassette	Construction of KIR1-Gateway	ccdB-F1	CTGCAGGTCGACTAGGCCTGTTATCCCTAACAAGT	ccdB-R1	TAGTTTAAACTCGGAATTATCGAACCACTTTGTAC
Gateway cassette	Construction of KIR1-Gateway	ccdB-F2	actagtgcggccgcctgcaggTCGACTAGGCCTGT	ccdB-R2	ctgtcaaacactgatagtttAAACTCGGAATTATC
**Complementation test**					
NPF7.2 promoter and gene	Construction of proNPF7.2:gNPF7.2-pENTR4	(pE4)-proNPF7.2-F	ggaaccaattcagtcgactgCACTTCAACTGCCGATCATCC	gNPF7.2-(pE4)-R	agctgggtctagatatctcgCAGACTTCCTCCTCTTCAGT
pENTR4	Linearization of pENTR4	pE4-InvF1	CGAGATATCTAGACCCAGCTTTCTTG	pE4-InvR1	CAGTCGACTGAATTGGTTCCCATG
proNPF7.2:gNPF7.2-pENTR4	Linearization of proNPF7.2:gNPF7.2-pENTR4	proNPF7.2-gNPF7.2-pE4-InvF	ATGGATCAAAAAGTTAGACAGTTTGAGG	proNPF7.2-gNPF7.2-pE4-InvR	AGATTCGATTTGGTGTAGAGATATACTC
Omega translational enhancer (Ω)	Ω fragment generation	(proNPF7.2)-Ω-(gNPF7.2)-st	ctctacaccaaatcgaatctACAATTACCAACAACAACAAACAACAAACAACATTACAATTACATTTACAATTACatggatcaaaaagttagaca	(proNPF7.2)-Ω-(gNPF7.2)-cp	tgtctaactttttgatccatGTAATTGTAAATGTAATTGTAATGTTGTTTGTTGTTTGTTGTTGTTGGTAATTGTagattcgatttggtgtagag
**NPF7.2 promoter-reporter analysis**					
NPF7.2 promoter	Construction of proNPF7.2-pENTR4	(pE4)-proNPF7.2-F	aaaagcaggctccaccatgCACTTCAACTGCCGATCATCC	proNPF7.2-(pE4)-R	ctgggtctagatatctcgaAGATTCGATTTGGTGTAGAGA
**NPF7.2 overexpression**					
NPF7.2 CDS	Construction of cNPF7.2-pENTR4	(pE4)-cNPF7.2-F1	ggaaccaattcagtcgactgATGGATCAAAAAGTTAGACAGTTTGAGG	cNPF7.2-(pE4)-R1	agctgggtctagatatctcgTCAGACTTCCTCCTCTTCAGT

Supplementary Table S1. Primers and synthetic DNA used in this study (*Continued*).

Target*	Use for	Forward primer		Reverse primer	
		Name	Sequence (5'-3')	Name	Sequence (5'-3')
**Direct transport assay**					
NPF7.2 CDS without stop codon	Construction of cNPF7.2ΔSTOP-pENTR4	(pE4)-cNPF7.2-F2	tgggaaccaattcagtcgacATGGATCAAAAAGTTAGACAGTTTGAGG	cNPF7.2-(pE4)-R2	agctgggtctagatatctcgAGACTTCCTCCTCTTCAGTTACG
pENTR4	Linearization of pENTR4	pE4-InvF1	CGAGATATCTAGACCCAGCTTTCTTG	pE4-InvR2	GTCGACTGAATTGGTTCCCATGGTGGA
cNPF7.2-GFP fusion	Construction of cNPF7.2-GFP-pENTR4	(pE4)-cNPF7.2-F1	ggaaccaattcagtcgactgATGGATCAAAAAGTTAGACAGTTTGAGG	GFP-(pE4)-R	agctgggtctagatatctcgTTACTTGTACAGCTCGTCCATG
**Semi-quantitative and quantitative expression analysis**					
NPF7.2	Semi-quantitative PCR	NPF7.2-QF1	GAGTTGAAGATTCGCCGTGT	NPF7.2-QR1	CCAGTTTATATCACAGAACAAGCTCAG
NPF7.2	Quantitative PCR	NPF7.2-QF2	GATGTGCATGGCTGGATTCC	NPF7.2-QR1	CCAGTTTATATCACAGAACAAGCTCAG
NPF7.2	Quantitative PCR	NPF7.2-QF3	TTCAACAGCCAAGCACCAAC	NPF7.2-QR2	AAACCACGAAATCAGCAGCG
PDR9§	Quantitative PCR	PDR9-QF	GTCTTGGACACTCAACGGGT	PDR9-QR	ATCTTGCAACCGTCGTGGAT
F6'H1§	Quantitative PCR	F6'H1-QF	TGATATCTGCAGGAATGAAACG	F6'H1-QF	GGGTAGTAGTTAAGGTTGACTC
BGLU42§	Quantitative PCR	BGLU42-QF	ATGGCCTGGGAACTGAAGTC	BGLU42-QF	ATTTGTCCAACCTCCGATTG
PP2AA3§	Quantitative PCR	PP2AA3-QF	TAACGTGGCCAAAATGATGC	PP2AA3-QR	GTTCTCCACAACCGCTTGGT

*Gene symbol provided by TAIR (http://www.arabidopsis.org/) except for T-DNA.

†Primer pairs were designed by the T-DNA Primer Design (http://signal.salk.edu/tdnaprimers.2.html).

§Primer pairs were designed by Gao *et al.* (2020).

Green lowercase letters indicate BbsI overhangs.

Blue lowercase letters indicate overlapping sequence complementary to ends of the linearized vector.

Reference

Dinneny JR, Long TA, Wang JY, Jung JW, Mace D, Pointer S, Barron C, Brady SM, Schiefelbein J, Benfey PN. 2008. Cell identity mediates the response of Arabidopsis roots to abiotic stress. *Science* **320**(5878): 942-945.

Gao F, Robe K, Bettembourg M, Navarro N, Rofidal V, Santoni V, Gaymard F, Vignols F, Roschzttardtz H, Izquierdo E, et al. 2020. The Transcription Factor bHLH121 Interacts with bHLH105 (ILR3) and Its Closest Homologs to Regulate Iron Homeostasis in Arabidopsis. *Plant Cell* **32**(2): 508-524.
